# A versatile and highly efficient method for scarless genome editing in *Escherichia coli* and *Salmonella enterica*

**DOI:** 10.1186/1472-6750-14-84

**Published:** 2014-09-25

**Authors:** Juhan Kim, Anthony M Webb, Jamie P Kershner, Stephen Blaskowski, Shelley D Copley

**Affiliations:** 1 Department of Molecular, Cellular, and Developmental Biology, University of Colorado Boulder, Boulder, CO 80309, USA; 2 Cooperative Institute for Research in Environmental Sciences, University of Colorado Boulder, Boulder, CO 80309, USA

**Keywords:** Genome editing, I-SceI, *Escherichia coli*, *Salmonella enterica*

## Abstract

**Background:**

Recently developed methods for genome editing in bacteria take advantage of the introduction of double-strand breaks by I-SceI in a mutation cassette to select for cells in which homologous recombination has healed the break and introduced a desired mutation. This elegantly designed method did not work well in our hands for most genes.

**Results:**

We corrected a mutation in the gene encoding I-SceI that compromised the function of a previously used Red helper plasmid. Further, we found that transcription extending into the mutation cassette interferes with cleavage by I-SceI. Addition of two transcription terminators upstream of the cleavage site dramatically increases the efficiency of genome editing. We also developed an improved method for modification of essential genes. Inclusion of a segment of the essential gene consisting of synonymous codons restores an open reading frame when the mutation cassette is integrated into the genome and decreases the frequency of recombination events that fail to incorporate the desired mutation. The optimized protocol takes only 5 days and has been 100% successful for over 100 genomic modifications in our hands.

**Conclusions:**

The method we describe here is reliable and versatile, enabling various types of genome editing in *Escherichia coli* and *Salmonella enterica* by straightforward modifications of the mutation cassette. We provide detailed descriptions of the methods as well as designs for insertions, deletions, and introduction of point mutations.

## Background

A facile and completely general method for modification of any genomic sequence of interest in bacteria would be of enormous value for a wide range of applications, including construction of strains for metabolic engineering, studies of the effects of mutations acquired during adaptive evolution, and construction of strains for molecular biology studies that require chromosomal modification of genes such as allele replacement or epitope tagging.

The most frequently used techniques for genetic modification of *Escherichia coli* and *Salmonella enterica* are the two-step recombination method [1,2] and the ‘gene gorging’ method [3]. Both methods use the bacteriophage λ recombination system to promote gene replacement [4] in strains with a low intrinsic recombination rate. For this purpose, Datsenko and Wanner [1] developed the Red helper plasmid, pKD46, and the Blattner lab developed two derivatives, pACBSR [3] and pK-HT [2]. Some variations on these commonly used methods use a λ Red recombinase encoded in the genomic DNA [5-7] rather than a Red helper plasmid.

The two-step recombination method uses a selection marker to identify cells in which a mutation cassette composed of two homologous recombination fragments surrounding a selection marker has been integrated into the target site. Subsequently, the selection marker can be removed by FLP recombinase [1] or by cleavage with the I-SceI meganuclease followed by homologous recombination using the host’s RecA system [2]. Use of FLP recombinase leaves a short FRT sequence as a scar. Use of the host’s RecA system after double-strand breakage by I-SceI leaves no scar at the end. In contrast, the gene gorging method relies upon screening to identify colonies in which a desired genetic change has been introduced by recombination of a mutation cassette into the genome. It also leaves no scar, but can be inefficient without the use of a special recombination strain [8] or a specially designed synthetic DNA with chemically modified bases [9]. These techniques have been used extensively and modified to enable deletions, insertions, and epitope tagging [5,10-18].

In our hands, genome editing by both the two-step recombination method and gene gorging was highly inefficient in most cases. We focused our attention on the two-step recombination method because of the advantage of employing selection to identify cells in which the mutation cassette has been integrated into the genome. We identified and corrected three mutations that limit the efficacy of a previously used helper plasmid and streamlined the procedure so that it can be carried out with higher efficiency and fewer experimental manipulations. However, we still observed a wide range of efficiencies depending on the identity of the target gene.

We discovered that the intransigence of certain genes was due to active transcription that continued into the inserted mutation cassette and prevented cleavage at the I-SceI site. Difficulties in modifying actively transcribed genes present an important limitation to the use of genome editing procedures. Inclusion of two successive terminator sequences in front of the I-SceI cleavage site obviated this problem and resulted in highly reliable chromosomal manipulation.

Modification of essential genes is also problematic using the two-step procedure because the first step results in interruption of the target gene by the mutation cassette. A solution to this dilemma is to include in the mutation cassette an extra fragment of the target gene that restores an open reading frame when the mutation cassette is integrated into the genome. However, since homologous recombination can occur anywhere within this extended extra fragment, many recombination events will fail to integrate the small region encoding the desired mutation into the genome. We addressed this problem by using a fragment of the target gene composed of synonymous codons to prevent homologous recombination within this extra fragment.

**Figure 1:**
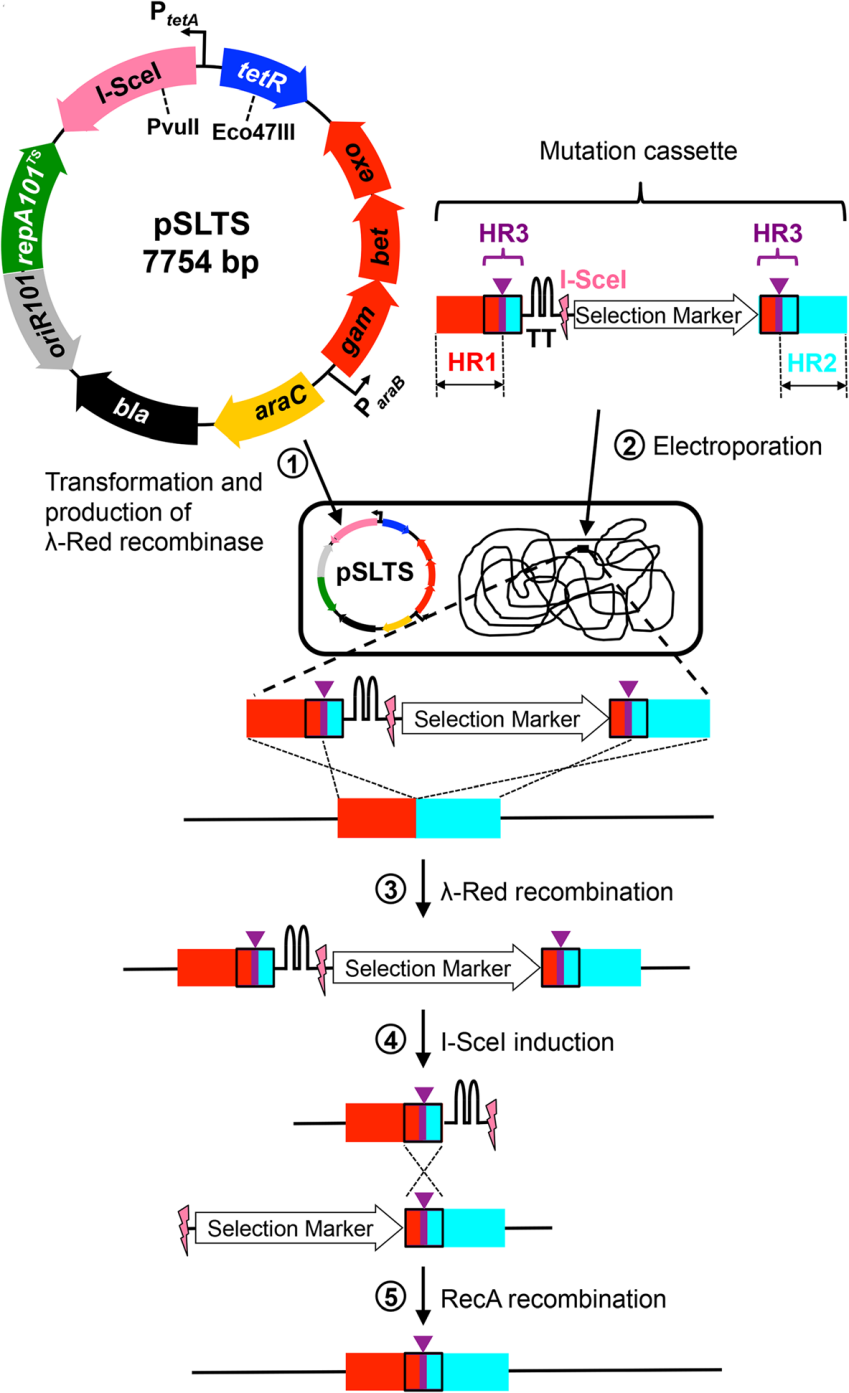
**The general strategy for scarless genome editing.** HR, homology region. HR1 and HR2 are identical to the target site in the genome. The purple bar and wedge in HR3 indicate the mutation that is to be introduced.

Our optimized procedure is summarized in Figure 1. The new helper plasmid pSLTS encodes I-**S**ceI under the control of the anhydrotetracycline-inducible *tetA* promoter and the **L**ambda Red recombinase genes under the control of the arabinose-inducible *araB* promoter, and carries a **T**emperature-**S**ensitive origin of replication. (Descriptions of all plasmids used in this work are provided in Table 1). The mutation cassette consists of a fragment containing 100 bp homology regions (HR1 and HR2) that target the cassette to the desired chromosomal location. HR3 provides the region in which homologous recombination can occur after I-SceI cleavage of the genomic DNA. Two HR3 sequences flank a sequence encoding a double transcriptional terminator (depicted as the double hairpin, ‘TT’, in Figure 1) in front of the I-SceI recognition site and a selection marker. The first step in the procedure is introduction of the helper plasmid pSLTS into the target strain. Overexpression of the λ Red recombinase is then induced by L-arabinose (Figure 1, Step 1). The mutation cassette is then introduced into cells carrying the helper plasmid by electroporation (Figure 1, Step 2). Integration of the mutation cassette into the target location is accomplished by the λ Red recombinase (Figure 1, Step 3). Cells in which the mutation cassette has been integrated into the genome are selected on plates containing the antibiotic for which the selection cassette confers resistance. Subsequently, these cells are spread onto plates containing ampicillin (to maintain the helper plasmid) and anhydrotetracycline (to induce expression of I-SceI) (Figure 1, Step 4). Most of the cells do not survive the double-strand break introduced by I-SceI. However, in a minority of cells, homologous recombination between the HR3 sequences enables survival and accomplishes the desired chromosomal modification (Figure 1, Step 5). Here we describe the development and validation of this method with applications in both *E. coli* and *Salmonella*.

**Table 1:** Plasmids used in this work

**Plasmids**	**Characteristics**	**Reference**
pSLTS*	ori SC101(Ts) Amp^r^; P_araB_ for λ-Red; P_tetR_ for I-SceI	This work
pHA_1887_	1887 bp fragment of pUC19 that contains the replication origin and the *bla* gene conferring ampicillin resistance	
pT2SC*	Amp^r^ Cm^r^; a template plasmid for amplification of a selection cassette that includes two transcription terminator elements, an I-SceI site and a chloramphenicol resistance gene ligated into pHA_1887_.	This work
pT2SK*	Amp^r^ Kan^r^; a template plasmid for amplification of a selection cassette that includes two transcription terminator elements, an I-SceI site and a kanamycin resistance gene ligated into pHA_1887_.	This work
pT2ST*	Amp^r^ Trim^r^; a template plasmid for amplification of a selection cassette that includes two transcription terminator elements, an I-SceI site and a trimethoprim resistance gene ligated into pHA_1887_.	This work
pT2SCb*	Amp^r^ Cm^r^; a template plasmid for amplification of a selection cassette that includes two transcription terminator elements, an I-SceI site and a modified chloramphenicol resistance gene that provides a bacterial consensus ribosomal binding site at the 3’-end ligated into pHA_1887_.	This work
pASC	Amp^r^ Cm^r^; a template plasmid for amplification of a selection cassette that includes an I-SceI site and a chloramphenicol resistance gene ligated into pHA_1887_.	This work
pASK	Amp^r^ Kan^r^; a template plasmid for amplification of a selection cassette that includes an I-SceI site and a kanamycin resistance gene ligated into pHA_1887_.	This work
pAST	Amp^r^ Trim^r^; a template plasmid for amplification of a selection cassette that includes an I-SceI site and a trimethoprim resistance ligated into pHA_1887_.	This work
pTSC	Amp^r^ Cm^r^; a template plasmid for amplification of a selection cassette that includes a transcription terminator, an I-SceI site and a chloramphenicol resistance gene ligated into pHA_1887_.	This work
pTSK	Amp^r^ Kan^r^; a template plasmid for amplification of a selection cassette that includes a transcription terminator, an I-SceI site and a kanamycin resistance gene ligated into pHA_1887_.	This work
pTST	Amp^r^ Trim^r^; a template plasmid for amplification of a selection cassette that includes a transcription terminator, an I-SceI site and a trimethoprim resistance gene ligated into pHA_1887_.	This work
pKD46	ori SC101(Ts) Amp^r^; P_araB_ for λ-Red	[1]
pK-HT	ori SC101(Ts^−^) Amp^r^; P_rha_ for λ-Red; P_tetR_ for I-SceI	[2]

*These plasmids have been deposited to the addgene repository and are freely available for academic use.

## Results and discussion

### Construction of an optimized helper plasmid

The ideal helper plasmid for two-step scarless chromosomal modification would have three characteristics: 1) genes encoding the λ-Red recombinase to increase the frequency of successful integration of the mutation cassette by recombination; 2) a gene encoding I-SceI; and 3) a temperature-sensitive origin of replication to facilitate removal of the helper plasmid after recombination of the mutation cassette into the genome. Further, the λ-Red recombinase genes and the gene encoding I-SceI should be inducible under different conditions. Three plasmids with these characteristics, pKDTS [2], pWRG99 [14] and pRedI [18], have been reported.

**Figure 2:**
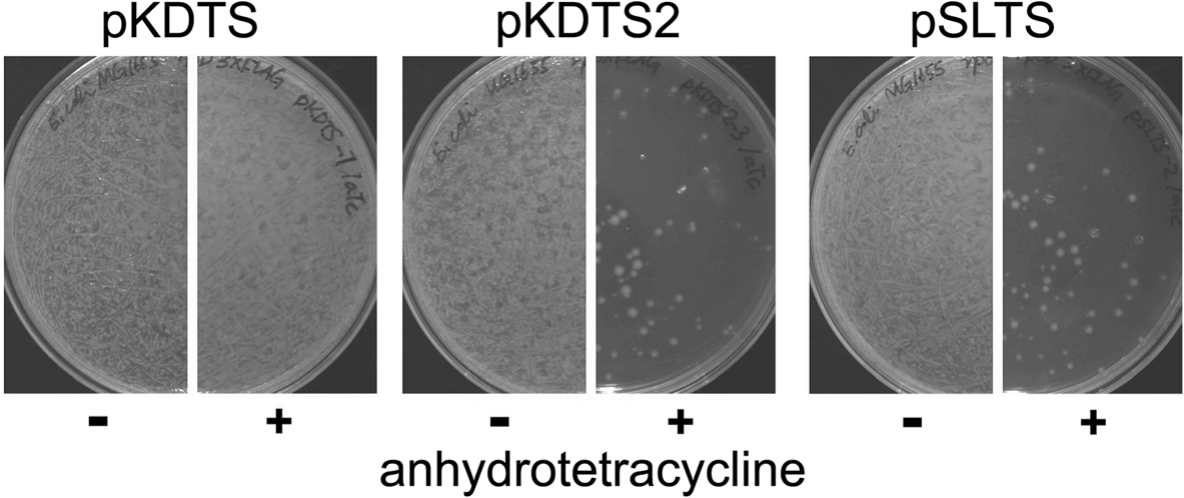
**Comparison of the performance of helper plasmids in enabling I-SceI cleavage of a target site.** Individual colonies of *E. coli* K-12 MG1655 cells containing the helper plasmid and into which a mutation cassette designed to introduce a C-terminal 3xFLAG tag on RpoD had been integrated were spread on agar plates containing LB Miller medium and 100 μg/mL ampicillin. Plates on the right half of each set contained 100 ng/ml anhydrotetracycline to induce expression of I-SceI. Successful cleavage of the mutation cassette results in a diminution of the number of viable colonies.

We used pKDTS as a helper plasmid to introduce a sequence encoding a 3 × FLAG tag at the 3’-end of *rpoD* in *E. coli*. We re-constructed this plasmid as previously described by inserting the 1407 bp Nco1 fragment encoding P_tet_-*I-sceI* from pK-HT [2] into the NcoI site of pKD46 [2]. We found that pKDTS effectively promoted recombination of the mutation cassette into the genome, as judged by growth of colonies on medium containing the antibiotic for which a resistance gene was carried on the mutation cassette. However, cleavage at the I-SceI site in the mutation cassette by I-SceI was extremely inefficient. After induction of I-SceI expression by anhydrotetracycline, only a small proportion of cells should survive; these cells have healed the double strand break introduced by I-SceI by a homologous recombination that removes the selection cassette. In this experiment, when 36 colonies were spread individually onto plates in the absence and presence of anhydrotetracycline, no difference in the number of colonies was observed (see Figure 2), suggesting that the intended cleavage of the genome by I-SceI was ineffective.

We discovered three mutations in the region of pKDTS derived from pK-HT. One of these, which results in replacement of 5 amino acid residues at the N-terminus of I-SceI with a single amino acid (MH*MKNIK*➜MH*Q*), had been detected previously [2]. Two other mutations resulted in missense changes of Asn11 to Ser and Lys33 to Arg in TetR (AAC➜AGC and AAG➜AGG, respectively). All three mutations were present in the pK-HT plasmid from which the fragment containing genes for I-SceI and TetR was taken. Any or all of these mutations might have been responsible for the failure of the I-SceI cleavage step. After correction of these three mutations, the new helper plasmid, pSLTS, was successfully used to introduce a sequence encoding a 3 × FLAG tag at the 3’-end of *rpoD* into *E. coli* (see Table 2 and Figure 2). The frequency of viable colonies after induction of I-SceI expression was 3.6 (±1.5) × 10^−5^, attesting to the efficiency of the I-SceI cleavage step. Among eight colonies that lost the selection cassette after induction of I-SceI expression, all had the correct insertion (Table 2). pKDTS2, a helper plasmid in which only the mutation in I-SceI had been corrected, also successfully promoted recombination of the mutation cassette into the genome and showed the expected diminution in the number of colonies after the induction of I-SceI expression (Figure 2). However, only four of eight colonies that had lost the antibiotic resistance marker had the correct insertion (Table 2). The remaining four colonies were wild type.

**Table 2:** **Comparison of the performance of helper plasmids in enabling introduction of a sequence encoding a C-terminal 3XFLAG tag on RpoD in *E. coli *** **K-12 MG1655**

**Helper plasmid**	**pKDTS2**	**pSLTS**
Fraction of colonies that lost the selection marker after double-strand cleavage of the mutation cassette by I-SceI	14/20	17/20
Fraction of colonies that had lost the selection marker for which a fragment of the correct size was amplified by colony PCR using primers flanking the gene of interest	4/8	8/8
Fraction of colonies for which the correct PCR product was obtained that contained the desired mutation	4/4	8/8

Scarless deletion of *thrB* in *E. coli* K-12 MG1655 and *argC* in *S. enterica* SL1344 was easily accomplished using pSLTS (Additional file 1: Table S1). Again, all of the colonies that had lost the selection cassette after induction of I-SceI expression had the intended deletion. The general design of constructs for both insertion and deletion of sequences is diagrammed in Additional file 1: Figure S1.

### Colony purification after introduction of the mutation cassette improves the efficiency of recovery of mutant strains

The recovery of colonies containing the desired mutation can be improved by re-streaking colonies obtained after introduction of the mutation cassette onto a second set of plates containing LB and ampicillin (LBA) and the antibiotic for which the mutation cassette confers resistance. Without this colony purification step, we routinely found some wild type colonies among those that grew on agar plates containing LB, ampicillin and anhydrotetracycline (LBAaTc). For example, in the experiment in which we introduced a sequence encoding a C-terminal 3 × FLAG tag on RpoD in *E. coli* K-12 MG1655 using pSLTS as a helper plasmid, 50% of the colonies obtained on LBAaTc agar plates were wild type when colony purification was not performed after integration of the mutation cassette. In contrast, no wild type cells were obtained when colony purification was performed (Table 2). When we attempted the same genetic modification in *S. enterica*, all of the 100 colonies tested after induction of I-SceI were wild type when colony purification was not done, while all of the 8 colonies tested had the desired modification when colony purification was performed. Apparently some wild type cells were able to survive in the vicinity of colonies that had integrated the mutation cassette and were able to detoxify the antibiotic in their immediate environment.

### Manipulation of highly transcribed genes requires an additional element in the selection cassette

**Figure 3:**
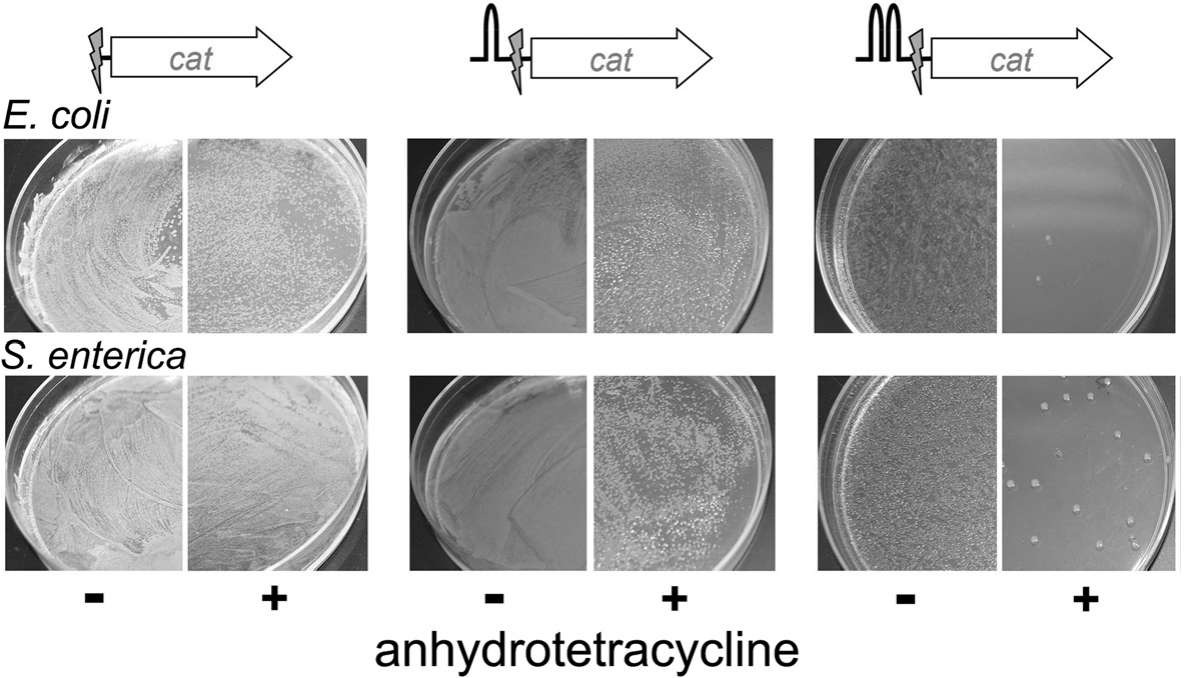
**Terminator elements up-stream of the I-SceI cleavage site enhance the efficiency of I-SceI cleavage.** One or two terminator elements were introduced ahead of the I-SceI cleavage site in mutation cassettes designed to introduce a sequence encoding a C-terminal 3 × FLAG tag on GapA in *E. coli* K-12 MG1655 and *S. enterica* Typhimurium SL1344. The efficiency of double-strand break formation by I-SceI was determined by spreading cells on agar plates containing LB Miller medium and 100 μg/mL ampicillin in the presence or absence of 100 ng/ml anhydrotetracycline.

The two-step recombination method using pSLTS worked well in many cases, including the insertion of a sequence encoding a 3 × FLAG tag at the 3’-end of *rpoD* discussed above. However, we consistently failed to generate successful mutants when we attempted to introduce point mutations into *rpe* and *aceE* in *E. coli*, to delete an intergenic region between *thrL* and *thrA* and a small region in *pta* in *E. coli*, and to introduce a sequence encoding a 3XFLAG-tag at the C-terminus of GapA in *E. coli* and at the C-termini of GapA and FbaA in *S. enterica*. In each case, the mutation cassette was successfully integrated into the genome in the first step. However, spreading cells onto plates containing anhydrotetracycline to induce expression of I-SceI did not result in the expected decrease in the number of colonies due to introduction of a double-strand break (Figure 3). This result suggested that I-SceI failed to cleave at its target site in the inserted mutation cassette. We hypothesized that active transcription initiated at the promoter upstream of the target gene might extend into the mutation cassette and interfere with binding of I-SceI to its target site. To test this hypothesis, we introduced one or two terminator sequences upstream of the I-SceI site. We used the two most-efficient artificial terminators in the iGEM parts collection (http://parts.igem.org/Terminators/Catalog). When only one terminator element (BBa_B1006, 99% efficiency) was present before the cleavage site, only a slight decrease in the number of colonies was observed after induction of I-SceI, suggesting that cleavage at the I-SceI site was still inefficient. Insertion of two terminator elements (BBa_B1006 and BBa_B1002, 99 and 98% efficiencies, respectively) resulted in a dramatic increase in cleavage efficiency, evidenced by the substantial decrease in the number of colonies obtained when cells were spread on plates containing anhydrotetracycline (Figure 3). In both *E. coli* and *S. enterica*, it was possible to identify a correct mutant by screening fewer than a dozen colonies when the selection cassette contained a double terminator element, whereas it was necessary to screen forty colonies to find a correct mutant when the selection cassette contained a single terminator element (Table 3).

**Table 3:** **Effect of terminator elements ahead of the I-SceI cleavage site on introduction of sequence encoding a C-terminal 3XFLAG tag on GapA in *E. coli *** **K-12 MG1655 and *S. enterica *** **Typhimurium SL1344**

	***E. coli *** **K-12 MG1655**			***S. enterica *** **Typhimurium SL1344**		
Number of terminators in front of the I-SceI recognition site	0	1	2	0	1	2
Fraction of colonies that lost the selection marker after double-strand cleavage of the mutation cassette by I-SceI	0/100	2/40	5/11	0/100	1/40	4/7
Fraction of colonies that lost the selection marker for which a fragment of the correct size was amplified by colony PCR using primers flanking the gene of interest	NA*	1/2	1/2	NA*	1/1	2/2
Fraction of colonies for which the correct PCR product was obtained that contained the desired mutation	NA*	1/1	1/1	NA*	1/1	2/2

*NA, not applicable.

### Introduction of mutations in essential genes

Introduction of mutations in essential genes is difficult because the typical strategy for initial integration of a mutation cassette into the genome disrupts the target gene. We have designed a modification of this strategy that enables efficient manipulation of essential genes.

*frr* and *ppa* are essential for growth of *E. coli* K-12 BW25113 in rich media such as LB [19]. *frr* encodes a ribosome recycling factor and *ppa* encodes an inorganic pyrophosphatase. These genes were previously used to demonstrate a method for introduction of an amber stop codon that could subsequently be used in conjunction with a suppressor tRNA to enable condition-specific disruption of translation [2]. We targeted these genes to facilitate comparison of our approach and the previously published approach.

**Figure 4:**
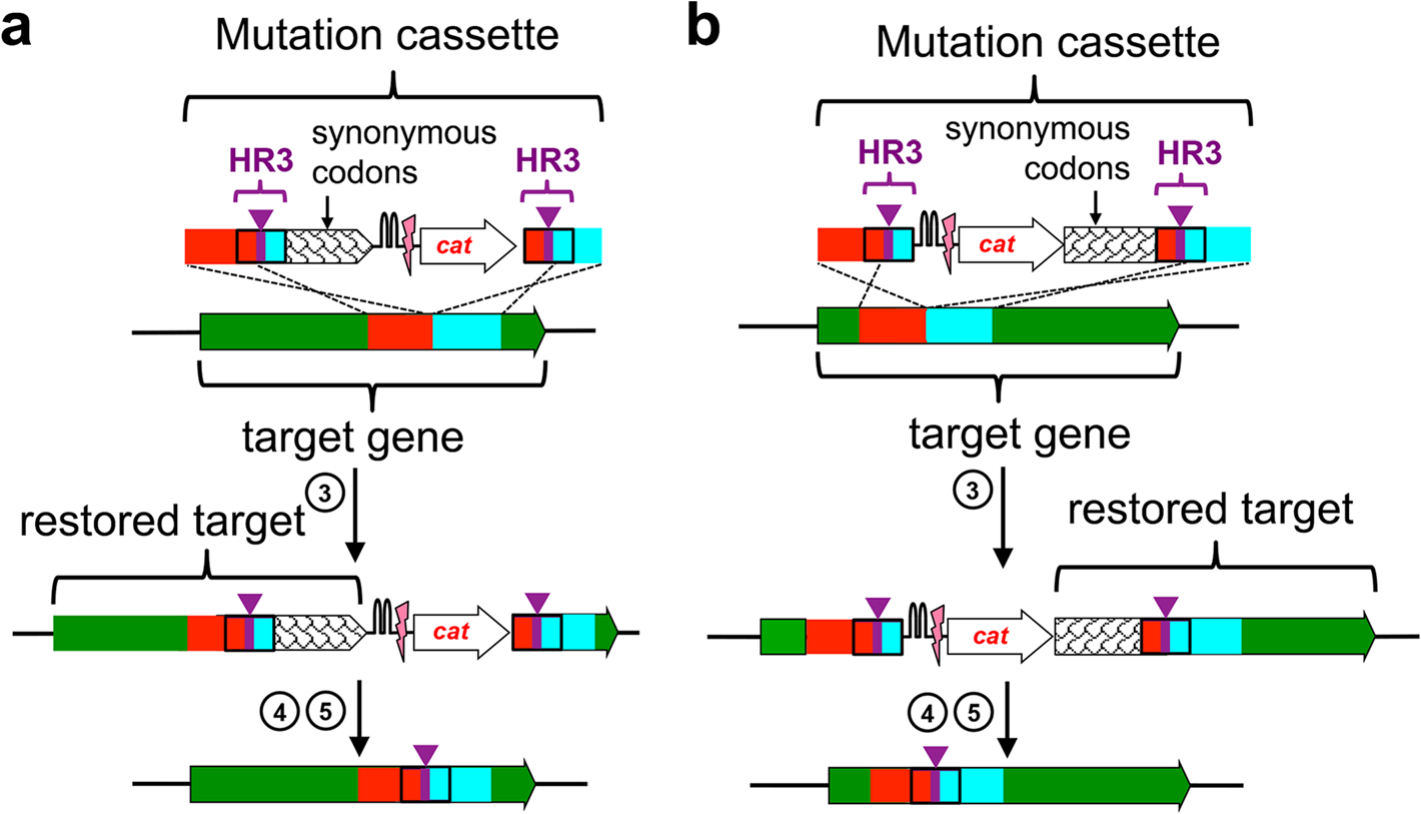
**A gene fragment with synonymous codons maintains function of an essential gene during editing.** An intact open reading frame can be reconstituted either under control of its own promoter **(a)** or under control of the constitutive promoter upstream of the selection marker (*cat*) preceding the target gene fragment **(b)**.

**Figure 5:**
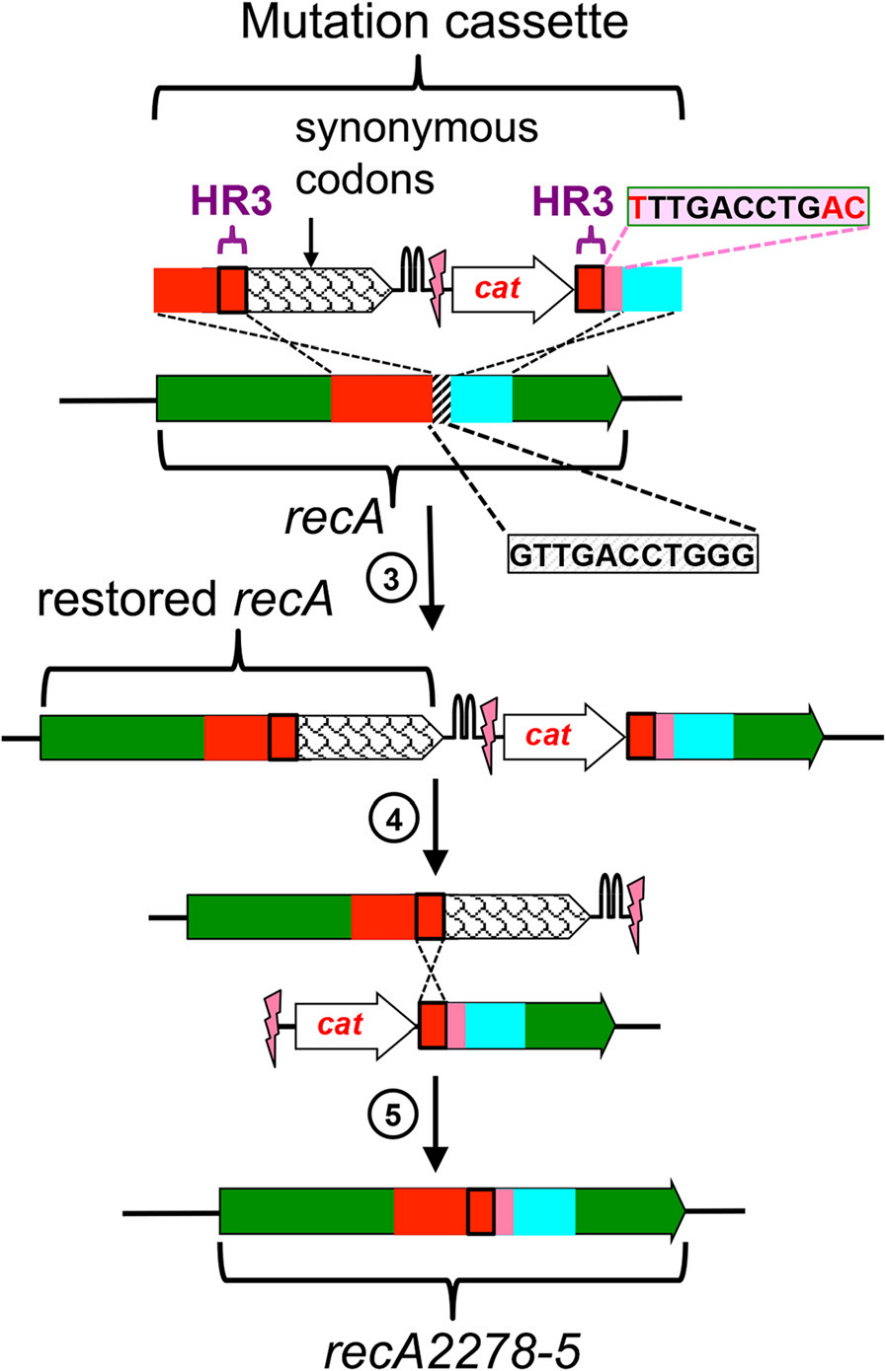
**Introduction of multiple point mutations into *recA *in *E. coli*.** The hatched area indicates the region to be replaced by the pink fragment of the mutation cassette.

We designed a mutation cassette in which HR3 contains a silent mutation of a codon for serine (AGC➔TCT or TCT➔AGC) in either the 5’-half or the 3’- half of *frr* and *ppa.* To maintain the function of the encoded proteins during the procedure, we added an additional fragment that encodes part of the target gene so that an intact open reading frame was reconstituted either under control of its own promoter (Figure 4a) or under control of the constitutive promoter upstream of the selection marker (*cat*) preceding the target gene fragment (Figure 4b) after integration of the mutation cassette into the genome. (The latter strategy would be optimal for editing of the first gene in an operon because it would avoid polar effects on downstream genes). Each codon of this additional fragment was replaced with a synonymous codon to produce a sequence that encodes the same protein but cannot recombine with the target gene in the genome. An alternative design (Figure 5 and further discussion below) restores a complete open reading frame for the wild type sequence of the essential gene, which resolves to a modified gene after double-strand cleavage and homologous recombination. In addition to maintaining the function of the target gene throughout the procedure, this strategy helps confine the sequences capable of recombination to a small window, thereby decreasing the frequency of recombination events that regenerate the wild type sequence (Additional file 1: Figure S2). Such recombination events may account for the variable frequency of integration of the amber stop codon into target genes in a previous report [2]. Because unproductive recombination events increase the amount of screening required to identify colonies with the desired mutation, the synonymous codon strategy significantly improves the efficiency of the procedure, which is of particular importance for endeavors requiring a large number of modifications.

We were able to introduce silent mutations into both the 5’ and 3’ halves of *frr* and the 3’ half of *ppa* using this approach (Table 4). The mutation cassette was successfully integrated in 100% of the colonies obtained after selection on agar plates containing the antibiotic for which the selection marker conferred resistance. We typically selected four colonies from these plates and spread them onto plates containing ampicillin with or without anhydrotetracycline to induce expression of I-SceI. Effective cleavage by I-SceI was evidenced by a dramatic reduction in the number of colonies on the plates containing anhydrotetracycline. Subsequently, we patched up to five colonies from each plate containing anhydrotetracycline onto plates in the absence and presence of the antibiotic for which the mutation cassette confers resistance. All of the colonies that lost the antibiotic resistance marker contained the desired mutation (Table 4).

**Table 4:** **Efficiency of introduction of silent mutations into essential genes in *E. coli *** **K-12 BW25113 using pSLTS as a helper plasmid**

**Target gene**	***frr***		***ppa***	
**length of gene (bp) affected amino acid residue**	**558 Ser33**		**531**	
Position of mutation (bp)	97-99	445-447 Ser149	109-111 Ser37	343-345 Ser115
Mutation	TCT➜AGC	AGC➜TCT	AGC➜TCT	AGC➜TCT
Efficiency of integration of the mutation cassette	100% (4/4)	100% (4/4)	67% (2/3)	100% (4/4)
Fraction of colonies that lost the selection marker after double-strand cleavage of the mutation cassette by I-SceI	2/7	3/6	4/10	9/20
Fraction of colonies that lost the selection marker for which a fragment of the correct size was amplified by colony PCR using primers flanking the gene of interest	2/2	3/3	2/2	4/4
Fraction of colonies for which the correct PCR product was obtained that contained the desired mutation	2/2	3/3	2/2	4/4

**Figure 6:**
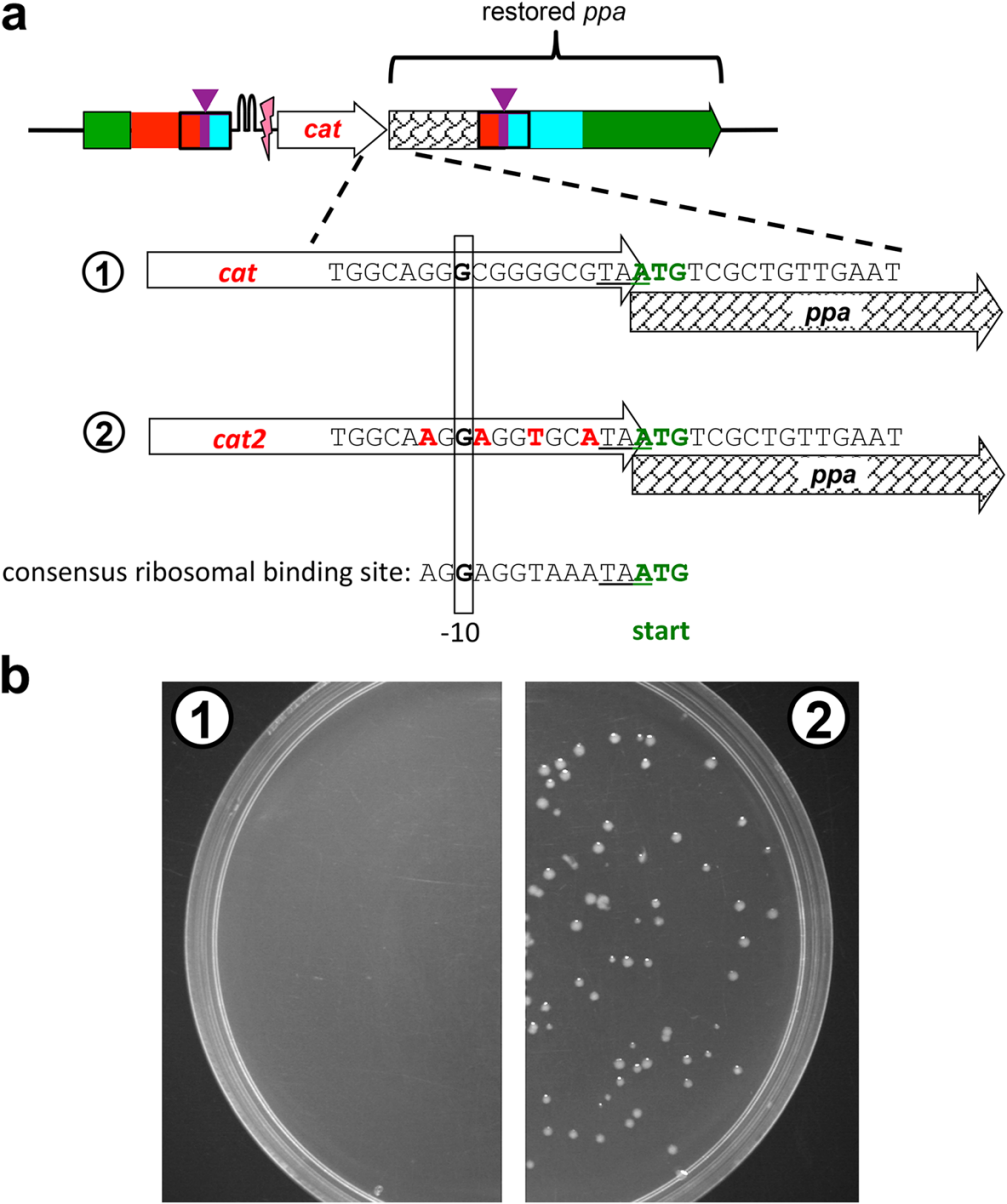
**Introduction of a ribosome binding site improves production of the protein encoded by the downstream gene. (a)** Four silent mutations at the 3’-end of *cat* were introduced to generate a more effective ribosomal binding site upstream of *ppa.* Sequences at the junction between *cat* and *ppa* are shown for the original (1) and modified (2) constructs. **(b)** Growth of cells on plates containing ampicillin (to force retention of the helper plasmid) and chloramphenicol (to confirm integration of the mutation cassette into the genome) after introduction of the mutation cassette by electroporation. The lack of colonies on the left indicates that construct 1 does not enable production of the essential protein Ppa. However, construct 2 allows growth, indicating that sufficient Ppa is produced.

We were initially unable to introduce a silent mutation in the 5’ half of *ppa* due to a failure to integrate the mutation cassette, suggesting that our construct did not enable production of sufficient Ppa to support growth. This failure might have been due to difficulties in transcription due to the dependence of *ppa* transcription on the constitutive promoter upstream of *cat*, although this was not an issue in the comparable experiment with *frr.* A more likely explanation is that translation of the synonymously coded fragment was sup-optimal. To explore this possibility, we introduced silent mutations in the *cat* gene upstream of the synonymous coding region of *ppa* in the mutation cassette to generate a ribosome binding site more closely resembling the bacterial consensus ribosome binding site [20]. This strategy was successful (see Figure 6), suggesting that a strong ribosome binding site upstream of an essential gene that includes synonymous codons may be required to produce sufficient protein for cell survival in some cases.

### λ-Red recombinase can be used to increase the efficiency of double-strand break repair

The synonymous codon strategy described above can be used to regenerate an open reading frame for either the mutant target gene (Figure 4) or the wild type target gene (Figure 5) after integration of the mutation cassette into the genome. The latter strategy is useful when it is desirable to maintain the wild type function of the gene until the final step. This was the case when we endeavored to introduce three mutations found in the *recA*2278*-5* allele [21] into *recA* in *E. coli* because active RecA is needed to repair the double-strand break introduced by I-SceI [2] in the second step of the genome editing procedure. Even though wild type RecA should have been available, we found that the frequency of loss of the selection marker after induction of I-SceI was low; only two out of 25 colonies patched to LBA agar plates lost resistance to the antibiotic (Additional file 1: Table S2). The reconstitution of the *recA* gene using synonymous codons may have resulted in a lower level of RecA. To counter this difficulty, we over-expressed the Red recombinase before inducing expression of I-SceI. It has previously been reported that Exo and Beta of the Red recombinase increase the efficiency of double-strand break repair by 100-fold in cells containing wild type RecA [22]. Indeed, overproduction of Red recombinase dramatically improved the efficiency of the mutagenesis; all of the 25 colonies that were tested after the I-SceI induction step had lost the selection marker (Additional file 1: Table S2), indicating that homologous recombination had occurred.

## Conclusions

Although impressive progress has been made in the past decade toward developing methods for introduction of specific genetic changes into the genomes of *E. coli* and *S. enterica*, the ease of performance and the efficiencies of even the best of these methods vary widely. We have identified the major impediments to the success of strategies that require creation of double-strand breaks by I-SceI followed by homologous recombination to accomplish genetic changes. In addition, we found that transcription initiated upstream of the mutation cassette apparently interferes with the ability of I-SceI to cleave at its recognition site. Introduction of two terminators upstream of the I-SceI site alleviates this problem.

Genetic modification of essential genes is particularly challenging because the function of the gene must be maintained throughout the procedure. We have shown that use of a mutation cassette in which synonymous codons restore the coding sequence of the gene after integration into the genome has the double benefit of maintaining function and limiting the region within which homologous recombination can take place, thereby increasing the proportion of crossovers that incorporate the desired change into the genome.

The method described here has enabled highly efficient modification of every gene we have targeted, including a number for which previously published strategies have been unsuccessful. The streamlined approach we have developed substantially decreases the time and effort required to generate genetically modified strains; we routinely generate desired mutants within one week. In addition, the approach is also highly versatile, allowing introduction of one or several point mutations, insertions and deletions into even difficult targets, using a common set of materials and procedures.

The method described in this paper compares favorably to the most recently developed methods for genome editing in *E. coli* and *S. enterica.* The CRISPR/Cas system developed for genome editing in eukaryotes has recently been applied to bacteria. This method is less convenient than the method described here because it requires construction of an editing fragment and a specific targeting RNA for each mutation to be introduced, whereas our two-step recombination method requires only a mutation cassette. Only two examples of successful gene editing have been reported in *E. coli*[7], so the general applicability of the system in bacteria is not yet proven.

A distinctly different method for accomplishing insertions, deletions and inversions in bacterial genomes using mobile group II introns and the Cre-*lox* system has been recently described [23]. This method is most useful for large-scale genomic changes, rather than smaller-scale changes, such as introduction of a point mutation or a sequence encoding a tag. Furthermore, this method requires initial disruption of a target gene, and cannot be used in its present form for essential genes.

Finally, a new two-plasmid method for scarless genome editing in *E. coli* has been reported by Yang et al. [24]. This method combines gene gorging with the use of a selection marker whose loss is an indication of introduction of the desired mutation into the genome. The first plasmid carries the genes for the Red recombinase and the I-SceI endonuclease, as well as a selection marker flanked by I-SceI sites and homology regions to target a particular genomic site. After integration of the selection marker, a second plasmid carrying a mutation cassette, again flanked by I-SceI sites, is introduced. After induction of I-SceI, cleavage at its recognition sites in the plasmid and the genome generates fragments that can recombine to integrate the mutation fragment into the genome. This procedure was shown to be very efficient in three cases. However, it is not well suited for editing of essential genes. The method was used to edit an essential gene (*metK*, which is essential for biosynthesis of S-adenosylmethionine). However, the procedure required a clever trick; initial replacement of *metK* with a gene encoding a transporter allowed uptake of S-adenosylmethionine and rendered MetK activity dispensable. This approach will not be applicable to most cases. Further, the Yang et al. method requires more time and experimental manipulation than the method described here.

## Methods

### Materials

*E. coli* K-12 BW25113 and K-12 MG1655 were obtained from the Nara Institute of Science and Technology and the American Type Culture Collection (ATCC), respectively. *S. enterica* Typhimurium SL1344 was obtained from the Detweiler laboratory at the University of Colorado Boulder. Chemically competent *E. coli* Mach1^TM^ cells (Life Technologies, Inc., Grand Island, NY) were used for all cloning procedures. Bacteria were grown in Miller’s modified Luria Broth (LB) or on Miller’s Luria agar medium (Research Products International Corp.) unless otherwise specified. The following antibiotics were added to media as required for selection: ampicillin, 100 μg/mL; streptomycin, 50 μg/mL; chloramphenicol, 20 μg/mL; kanamycin, 50 μg/mL when the *kan* resistance gene was present on a high-copy-number plasmid and for selection after integration of the mutation cassette into the target DNA, and 20 μg/mL for subsequent experiments after the *kan* resistance gene had been integrated into the genomic DNA; trimethoprim, 50 μg/mL when the resistance gene was present on a high copy-number plasmid or 25 μg/mL when the resistance gene was integrated into the genomic DNA. Q5 High-Fidelity DNA polymerase (New England Biolabs) and Phusion High-Fidelity DNA polymerase (New England Biolabs) were used for polymerase chain reactions. Primer sequences and descriptions of synthetic DNA fragments are provided in the Additional file 1.

### Construction of Red helper plasmids

pKD46 and pK-HT were obtained from the Blattner lab (University of Wisconsin-Madison). pKD46 encodes the λ Red recombinase genes under control of the *araB* promoter and a temperature-sensitive origin of replication. We re-constructed the previously described pKDTS by inserting the 1407 bp Nco1 fragment from pK-HT, which encodes I-SceI under control of the anhydrotetracycline-inducible *tetA* promoter, into the NcoI site of pKD46 [2].

We constructed pSLTS by correcting three mutations found in pKDTS. A 555 bp fragment containing the three mutations and encompassing 405 bp of the 5’-end of the *tetR* gene, 81 bp of the *tetA* promoter, and 69 bp of the 5’-end of the I-SceI gene was removed from pKDTS by digesting the plasmid with AfeI (Eco47III) and PvuII. The remaining 7187 bp fragment was purified by gel extraction. The 5’-phosphate was removed using Antarctic phosphatase (New England BioLabs). A 610 bp synthetic DNA fragment (gBlock1) (Integrated DNA Technologies, Coralville, IA) was used to replace the removed fragment. gBlock1 contains 405 bp of the 5’-end *tetR* fragment with two mutations corrected based upon the sequence of the *tetR* gene of transposon Tn10 (GenBank Accession number, J01830.1), 81 bp of the *tetA* promoter, and 81 bp of the corrected 5’-end of the gene encoding I-SceI, as well as 21 and 22 bp complementary to the 7187 bp pKDTS fragment at the 5’- and 3’-ends, respectively. gBlock1 was assembled with the 7187 bp pKDTS fragment to make pSLTS by the one-step enzymatic DNA assembly method [25] using the Gibson Assembly Master Mix (New England BioLabs). The same procedure was used to make pKDTS2 from the 7187 bp fragment of pKDTS and gBlock2, which encodes intact I-SceI but still contains two mutations in *tetR*. Correct construction of pSLTS, pKDTS2, and pKDTS was confirmed by sequencing the modified parts of the plasmids using sequencing primers pKDTS-F and pKDTS-R.

### Construction of selection cassette template plasmids

We constructed a set of template plasmids from which selection cassettes containing the I-SceI site and a positive selection marker can be amplified for subsequent incorporation into mutation cassettes. These plasmids were constructed by ligating a selection cassette containing an I-SceI recognition site followed by an antibiotic resistance gene into pHA_1887_ (see further below). The resulting plasmids, pASC, pASK and pAST, contain selection cassettes that confer resistance to chloramphenicol, kanamycin and trimethoprim, respectively. A second generation of template plasmids was constructed by adding sequences encoding one or two artificial transcription terminators upstream of the I-SceI site. These plasmids were assembled from pHA_1877_, a sequence containing one or two transcriptional terminators (STS or STTS), and a selection cassette (amplified from a pAS series plasmid using primers MarkerF and MarkerR), using the one-step enzymatic DNA assembly method [25]. The names of these plasmids indicate the number of terminators and the antibiotic for which they confer resistance. pTS series plasmids contain one terminator element, an I-SceI recognition site and a gene conferring resistance to chloramphenicol (pTSC), kanamycin (pTSK), or trimethoprim (pTST). pT2S series plasmids contain two terminator elements, an I-SceI recognition site and a gene conferring resistance to chloramphenicol (pT2SC and pT2SCb), kanamycin (pT2SK) or trimethoprim (pT2ST). Correct insertion of selection cassettes into pHA_1887_ was confirmed in each case by sequencing inserted elements using sequencing primers pHA.seq.F and pHA.seq.R.

Selection cassettes to be used for construction of mutation cassettes were amplified from selection cassette template plasmids using primers MF and MR for pTS and pT2S series plasmids (except for pT2SCb, for which MR2 was used instead of MR) and primers MarkerF and MR for pAS series plasmids. After treatment of the amplification mixture with DpnI to remove the template plasmid, amplified selection cassettes were gel-purified.

The following paragraphs describe the origin and assembly of the various pieces of the selection cassette template fragments.

pHA_1887_ is an 1887 bp fragment amplified from pUC19 using primers pHAFor and pHARev. pHA_1887_ contains the replication origin that confers high copy number and the *bla* gene that confers ampicillin resistance. PCR-amplified pHA_1887_ was treated with DpnI to remove the template plasmid and gel-purified.

Genes encoding antibiotic resistance were amplified using primers that introduced extra sequences for use in either PCR amplification of selection cassettes or in the genome editing procedure itself. The *cat* gene was amplified from pACYC184 using primers ISceIcatF and catR. ISceIcatF contains the I-SceI site recognition site followed by 18 bp of the 5’ end of *cat*. catR is complementary to the 3’ end of the *cat* gene. The *kan* gene was amplified from pACYC177 with primers ISceIkanF and kancat16R. ISceIkanF contains the I-SceI recognition site followed by 18 bp of the 5’-end of *kan*. kancat16R contains the last 16 bp of *cat* followed by the 3’-end of *kan*. (The 16 bp *cat* sequence was included so that a common primer (MR) could be used for subsequent amplification of selection cassettes containing either *kan* or *cat*). Amplified fragments containing either *cat* or *kan* were treated with DpnI to remove the template plasmid, gel-purified and ligated into the pHA_1887_ fragment using the Rapid DNA ligation kit (Thermo Fisher Scientific, Inc.) to make pASC and pASK, respectively.

gISceIdfrA is a synthetic DNA fragment designed to contain the I-SceI site followed by the 630 bp *dfrA* sequence (from the EZ-Tn5™ < DHFR-1 > (Epicentre)) and the 16 bp common primer binding sequence. Twenty bp sequences complementary to pHA_1887_ were included on each side. gISceIdfrA was assembled with pHA_1887_ to make pAST using the one-step enzymatic DNA assembly method [25].

gISceIcat2 is a synthetic DNA fragment designed to contain the I-SceI recognition site and a modified *cat* gene (*cat2*) in which the 3’-end sequence was modified to generate a sequence resembling the consensus bacterial ribosomal binding site [20].

The sequences of the terminator fragments STS and STTS (see Additional file 1: list of synthetic DNA fragments) were designed based upon iGEM parts. The sequence of STS, which contains one terminator flanked by two spacer elements, was patched together from spacer BBa_K259002, terminator BBa_B1006, and spacer BBa_B0040 parts. The sequence of STTS, which contains two terminator elements flanked by two spacer elements, was designed to minimize secondary structure in the spacer regions using UNAFold (Integrated DNA Technologies) and was patched together from part of spacer BBa_K259002, part of spacer BBa_B0040, terminator BBa_B1002, part of spacer BBa_B0040, terminator BBa_B1006, and part of spacer BBa_K259002. STS and STTS were prepared as minigenes (Integrated DNA Technologies) and were amplified by PCR using primers STSF and STSR. The amplification mixture was treated with DpnI to remove the template plasmid and the STS or STTS fragments were gel-purified.

### Construction of mutation cassette template plasmids

We constructed mutation cassette template plasmids to facilitate subsequent production of the mutation cassette by PCR amplification (see Additional file 1: Figure S3). Mutation cassettes contain a selection marker preceded by two transcriptional terminators and flanked by sequences homologous to the genomic target (see Figure 1). While 50 bp homology regions (HR1 and HR2) on each side of the selection marker are generally sufficient for homologous recombination into the genome, we typically obtained more colonies using 100 bp homology regions. Because the efficiency of recombination varies among genomic targets, we routinely use the longer 100 bp homology regions to ensure successful integration. The mutation cassette contains two identical regions (HR3) that span the desired genomic modification. The length of HR3 was 30–50 bp, sufficient for RecA-mediated recombination (see Step 5 in Figure 1), but short enough to minimize the frequency of recombination events that fail to incorporate the desired genetic change (see Additional file 1: Figure S2a).

Mutation template plasmids were assembled by mixing 5’- and 3’- mutation fragments (125 fmol each) with pHA_1877_, a linear fragment of pUC19 obtained by PCR (see Additional file 1: Information) (25 fmol), and a selection cassette encoding an antibiotic resistance gene (75 fmol). The volume of the mixture was adjusted to 10 μL with DNase-free water and then 10 μL of Gibson Assembly Master Mix (New England BioLabs) was added. After incubation for 1 hour at 50°C, 2 μL of the reaction mixture was introduced into chemically competent *E. coli* Mach1^TM^ cells following the manufacturer’s protocol. The transformed cells were spread on LB with ampicillin and the antibiotic to which the selection cassette conferred resistance. Typically, four colonies were selected for confirmation of the correct assembly of the mutation fragments and the correct sequence of the mutation cassette using primers pHA.seq.F and pHA.seq.R (see Additional file 1: Table S3).

Most mutation fragments used in the experiments described in this paper were obtained as synthetic double-stranded DNAs (gBlocks) from Integrated DNA Technologies, Inc., but mutation fragments can also be amplified from plasmid or genomic DNA. Generally, the 5’- mutation fragment contained approximately 200 bp of the target gene flanked by a sequence that overlaps the 3’-end of pHA_1887_ and a sequence that overlaps the 5’-end of a selection cassette. The 3’-mutation fragment also contains approximately 200 bp of the target gene flanked by a sequence that overlaps the 3’-end of the selection cassette and a sequence that overlaps the 5’-end of pHA_1887_. Both fragments contain a 30–50 bp region designated HR3 at the 3’-end of the 5’-mutation fragment and at the 5’-end of the 3’-mutation fragment. The desired mutation can be included in HR3 (Figure 1 and Figure 4), or immediately following HR3 (Figure 5).

Mutation cassettes were amplified from mutation template plasmids by PCR using appropriate primers (see Additional file 1: Figure S3). The template plasmid was removed by digestion with DpnI and the mutation cassette was purified by electrophoresis on a 1% TAE-agarose gel.

### Preparation of electrocompetent cells carrying a Red helper plasmid

A Red helper plasmid was introduced into *E. coli* K-12 BW25113 or *E. coli* K-12 MG1655 using the method of Chung et al. [26]. The plasmid (100 ng) was mixed with competent cells prepared from a 1 mL LB culture. A Red helper plasmid was introduced into electrocompetent *S. enterica* Typhimurium SL1344 cells by electroporation. (*S. enterica* Typhimurium SL1344 cells were rendered electrocompetent as described in Molecular Cloning [27]). The transformants were spread on LB Miller agar medium containing ampicillin (100 μg/mL). After overnight growth at 30°C, a single colony was used to inoculate 5 mL of LB Miller medium containing ampicillin (100 μg/mL) (LBA) for *E. coli* K-12 strains or ampicillin (100 μg/mL) and streptomycin (50 μg/mL) (LBAS) for *S. enterica* Typhimurium SL1344. After overnight incubation with shaking at 30°C, 1 mL of the culture was inoculated into 100 mL of LBA or LBAS in a 500 mL Erlenmyer flask. The culture was incubated with shaking for an hour at 30°C. L-Arabinose was added to a final concentration of 1 mM for *E. coli* K-12 BW25113 or 2 mM for *E. coli* K-12 MG1655 and *S. enterica* Typhimurium SL1344 to induce expression of the λ-Red recombinase. Incubation at 30°C with shaking was continued for 2–3 hours. When the OD_600_ of the culture reached 0.7-0.9, the cells were harvested by centrifugation at 4500 × g and washed twice with ice-cold 10% glycerol. The cells were resuspended in 10% glycerol and stored at −70°C before use.

### Genome editing procedure

A mutation cassette (50 to 100 ng) was mixed with 50 μL of electrocompetent cells carrying a Red helper plasmid on ice. After electroporation, 450 μL SOC [27] was added to the cells and the mixture was transferred to a 15 mL culture tube. Cells were incubated for 3 hours in a shaking incubator at 30°C and then spread onto plates containing LB plus ampicillin (LBA) for *E. coli* or LB plus ampicillin and streptocymcin (LBAS) for *S. enterica* Typhimurium SL1344 and an additional antibiotic (chloramphenicol (20 μg/mL), kanamycin (50 μg/mL), or trimethoprim (50 μg/mL)) as needed to select for cells in which the mutation cassette had been integrated into the genome. After overnight incubation at 30°C, four colonies were picked and streaked onto fresh selection plates. After incubation overnight at 30°C, one colony from each plate was suspended in sterile phosphate-buffered saline (PBS [27]). Because the frequency of double strand break repair is about 1 in 10^5^, it is only necessary to plate a small proportion of the cells in a colony in order to obtain enough colonies for the subsequent step. Aliquots of the cell suspensions were spread on plates containing LBA agar and LBA agar plus anhydrotetracycline (100 ng/mL) (LBAaTc). For *S. enterica*, the plates also contained streptomycin (50 μg/mL). In general, plating 50 to 100 μL of a cell suspension in which a single colony had been resuspended in 500 μL PBS resulted in several to tens of colonies on plates containing anhydrotetracycline. To confirm that double strand break repair had resulted in excision of the selection cassette, five to ten colonies from the LBAaTc plate were patched onto LB agar and onto LB agar supplemented with the antibiotic for which the mutation cassette provided resistance. (Streptomycin was also added for *S. enterica* Typhimurium SL1344). Ampicillin can be included in these plates to force retention of the helper plasmid if further rounds of mutations are planned. Colonies that grew on the LB agar plates but not on plates supplemented with the antibiotic were streaked onto fresh LB agar plates. After overnight incubation, a few individual colonies were used to prepare freezer stocks. The introduction of the desired mutation was verified by amplifying the target region by PCR and sequencing.

When necessary to increase the efficiency of double-strand break repair, expression of the Red recombinase was induced before expression of I-SceI. A single colony in which the mutation cassette had been integrated was resuspended in 50 μL of sterile DNase-free water. An aliquot (20 μL) was boiled at 100°C prior to amplification of the mutation cassette by PCR to confirm correct integration of the mutation cassette into the genome. The remaining aliquot (30 μL) was mixed with 120 μL of sterile PBS. Fifty μL of the suspended cells were spread on plates containing LBA or LBAaTc. The remaining 50 μL were inoculated into 1 mL of LBA in a 15 mL culture tube. The culture was incubated in a shaking incubator for an hour at 30°C. L-Arabinose was added to a final concentration of 1 mM for *E. coli* K-12 BW25113 or 2 mM for *E. coli* K-12 MG1655 and *S. enterica* to induce production of the Red recombinase*.* After 2 hours of further incubation at 30°C in a shaking incubator, 50 μL aliquots were spread on plates containing LBA or LBAaTc.

## Competing interests

The University of Colorado Boulder has filed a patent application related to this publication.

## Authors’ contributions

JK designed and carried out experiments and wrote the paper. AMW and SB carried out experiments. JPK designed the method for assembly of the mutation template plasmid. SDC supervised the experiments and wrote the paper. All authors read and approved the final manuscript.

## Supplementary Material

Additional file 1: Figure S1. Diagrams for design of mutation cassettes for insertion **(a)** and deletion **(b)**. **Figure S2.** Synonymous codon fragment increases the efficiency of genome editing of the essential target. **(a)** Homologous recombination of the mutation cassette into the target gene in step 3 can fail to introduce mutations in HR3 (purple band and wedge) of the 3’-end of the mutation fragment when the second cross-over occurs in either the green or red region before HR3. The consequence is regeneration of the wild type sequence during the second homologous recombination step (step 4). **(b)** The use of a synonymous codon fragment restricts the positions at which homologous recombination can take place in step 3, increasing the likelihood that the sequence that includes the mutations is properly integrated into the genome and that the desired mutation will be inserted into the target gene after double-strand cleavage (step 4) and homologous recombination (step 5). **Table S1.** Deletion of genes in *S. enterica* and *E. coli* using pSLTS. **Table S2**. λ Red recombinase contributes to double-strand break repair during chromosomal modification of *recA* in *E. coli* K-12 BW25113. **Table S3.** PCR and sequencing primers. **Table S4.** Components used to generate mutation cassettes. Supplemental List of synthetic DNA fragments. Click here for file
