# Supplementary material for: A versatile and highly efficient method for scarless genome editing in Escherichia coli and Salmonella enterica
Source: BMC Biotechnol. 2014 Sep 25;14:84. doi: 10.1186/1472-6750-14-84 (PMC4236582; doi:10.1186/1472-6750-14-84)
Supplement: Additional file 1: Figure S1. — Diagrams for design of mutation cassettes for insertion (a) and deletion (b). Figure S2. Synonymous codon fragment increases the efficiency of genome editing of the essential target. (a) Homologous recombination of the mutation cassette into the target gene in step 3 can fail to introduce mutations in HR3 (purple band and wedge) of the 3’-end of the mutation fragment when the second cross-over occurs in either the green or red region before HR3. The consequence is regeneration of the wild type sequence during the second homologous recombination step (step 4). (b) The use of a synonymous codon fragment restricts the positions at which homologous recombination can take place in step 3, increasing the likelihood that the sequence that includes the mutations is properly integrated into the genome and that the desired mutation will be inserted into the target gene after double-strand cleavage (step 4) and homologous recombination (step 5). Table S1. Deletion of genes in S. enterica and E. coli using pSLTS. Table S2. λ Red recombinase contributes to double-strand break repair during chromosomal modification of recA in E. coli K-12 BW25113. Table S3. PCR and sequencing primers. Table S4. Components used to generate mutation cassettes. Supplemental List of synthetic DNA fragments. [file 1472-6750-14-84-S1.docx]

**SUPPLEMENTARY FIGURES**

**
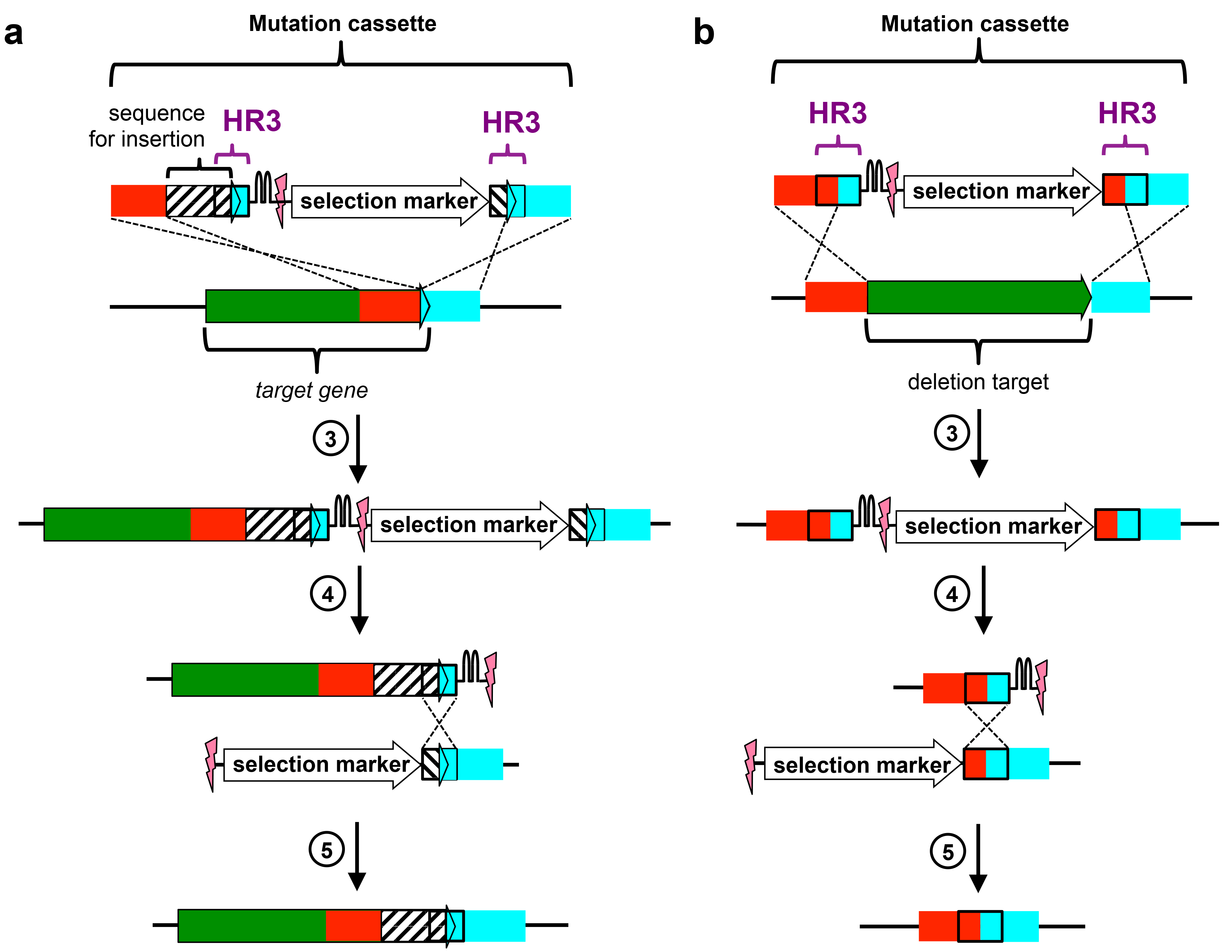
**

**Supplementary Figure 1.** Diagrams for design of mutation cassettes for insertion (a) and deletion (b).

**
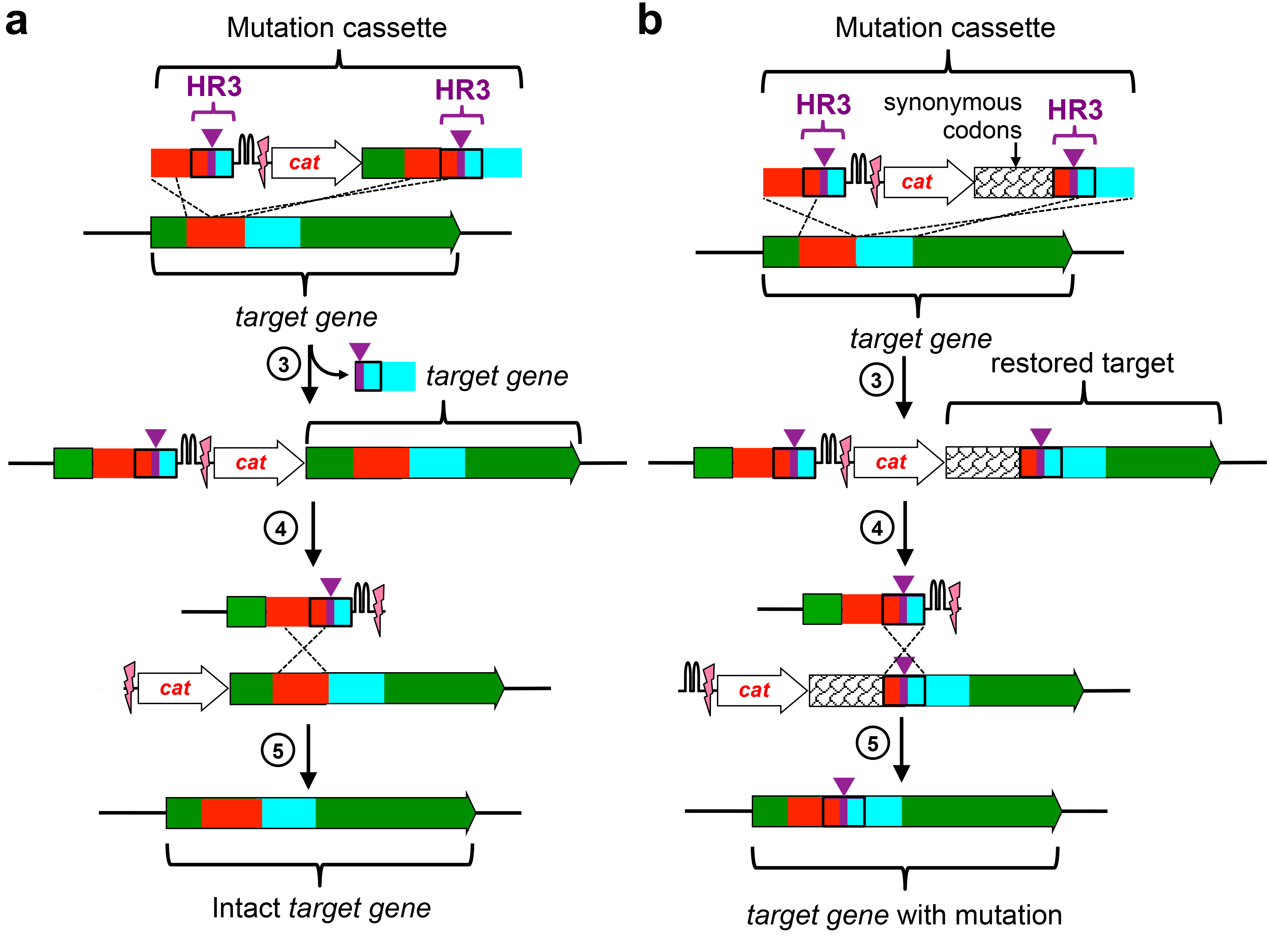
**

**Supplementary Figure 2.** Synonymous codon fragment increases the efficiency of genome editing of the essential target. (a) Homologous recombination of the mutation cassette into the target gene in step 3 can fail to introduce mutations in HR3 (purple band and wedge) of the 3’-end of the mutation fragment when the second cross-over occurs in either the green or red region before HR3. The consequence is regeneration of the wild type sequence during the second homologous recombination step (step 4). (b) The use of a synonymous codon fragment restricts the positions at which homologous recombination can take place in step 3, increasing the likelihood that the sequence that includes the mutations is properly integrated into the genome and that the desired mutation will be inserted into the target gene after double-strand cleavage (step 4) and homologous recombination (step 5).


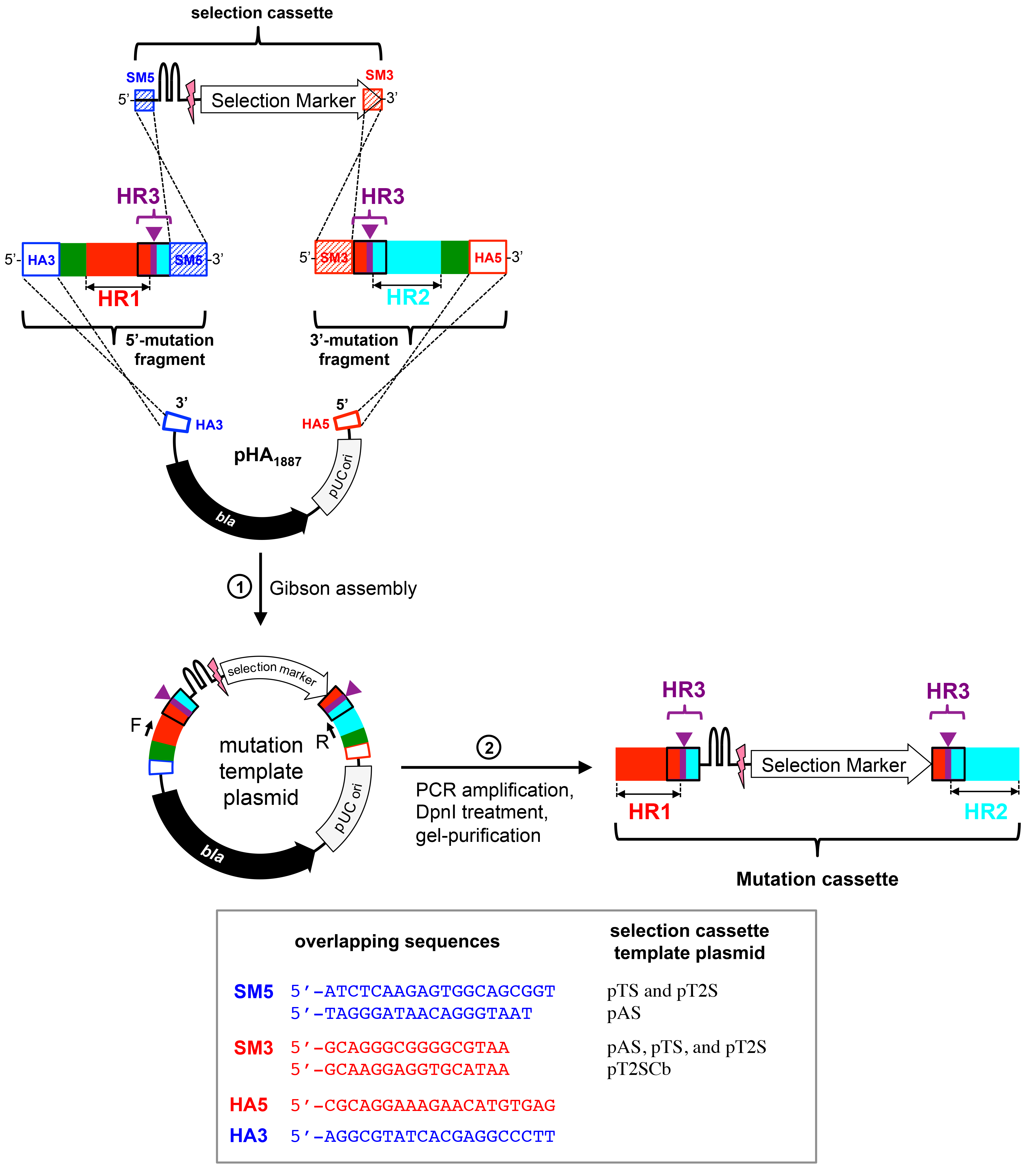


**Supplementary Figure 3.** Assembly of mutation cassette plasmids and their use for PCR amplification of mutation cassettes. Green fragments adjacent to HR1 and HR2 are target sequences added to provide the 200 bp length recommended for one-step assembly of the mutation template plasmid.

**SUPPLEMENTARY TABLES**

**Supplementary Table 1.** Deletion of genes in *S. enterica* and *E. coli* using pSLTS.

|  | Δ*thrB* in  *E. coli* K-12 MG1655 | Δ*argC* in  *S. enterica* Typhimurium SL1344 |
| --- | --- | --- |
| fraction of colonies patched from LBAaTc agar plates that had lost the selection marker after double-strand cleavage of the mutation cassette | 4/14 | 52/55 |
| fraction of colonies that lost the selection marker for  which a fragment of the correct size was amplified by  colony PCR using primers flanking the gene of  interest | 3/4 | 4/4 |
| fraction of colonies for which the correct PCR product was obtained that contained the desired mutation | 3/3 | 4/4 |

**Supplementary Table 2.** λ Red recombinase contributes to double-strand break repair during chromosomal modification of *recA* in *E. coli* K-12 BW25113.

| induction of λ Red recombinase | no | yes |
| --- | --- | --- |
| fraction of colonies patched from LBAaTc agar plates that had lost the selection marker after double-strand cleavage of the mutation cassette | 2/25 | 25/25 |
| fraction of colonies that lost the selection marker for  which a fragment of the correct size was amplified by  colony PCR using primers flanking the gene of  interest | 2/2 | 4/4 |
| fraction of colonies for which the correct PCR product was obtained that contained the desired mutation | 2/2 | 2/2 |

**Supplementary Table 3.** PCR and sequencing primers.

| Name | Sequence |
| --- | --- |
| pHA.seq.F | 5’-TATCAGGGTTATTGTCTCATGAGCG |
| pHA.seq.R | 5’-ACTTGAGCGTCGATTTTTGTGATGC |
| pKDTS-F | 5’-TAGGCGCAATCACTTTCGTCTACTC |
| pKDTS-R | 5’-TTGAGTGACATGCAAAGTAAGTATGATCTC |
| pHAFor | 5’-CGCAGGAAAGAACATGTG |
| pHARev | 5’-AAGGGCCTCGTGATACG |
| MF | 5’-ATCTCAAGAGTGGCAGC |
| MR | 5’-TTACGCCCCGCCCTGC |
| MR2 | 5’-TTATGCACCTCCTTGCCACTC |
| ISceIcatF | 5’-/5Phos/TAGGGATAACAGGGTAATCCTGGTGTCCCTGTTGAT |
| catR | 5’-/5Phos/TTACGCCCCGCCCTGCCA |
| ISceIkanF | 5’-/5Phos/TAGGGATAACAGGGTAATCTGATCCTTCAACTCAGC |
| kancat16R | 5’-/5Phos/TTACGCCCCGCCCTGCTTAGAAAAACTCATCGAGCATC |
| STSF | 5’-AGGCGTATCACGAGGCCCTTA |
| STSR | 5’-ATTACCCTGTTATCCCTAGC |
| MarkerF | 5’-TAGGGATAACAGGGTAATC |
| MarkerR | 5’-CTCACATGTTCTTTCCTGCGTTACGCCCCGCCCTGC |
| rpoD.ec.F | 5’-CGACGTTACCCGCGA |
| rpoD.ec.R | 5’-GCTGGTAGTGCGTGG |
| gapA.ec.F | 5’-TATCGCTCTGAACGACAACT |
| gapA.ec.R | 5’-TTCTTAATCATGACGCAGTC |
| gapA.st.F | 5’-CATCGCGCTGAACGAC |
| gapA.st.R | 5’-CATAGCCAACACACCTGC |
| frr33.silent.F | 5’-GTAACGTGATTAGCGATATC |
| frr33.silent.R | 5’-TTTTCAGTGTACGGGAATCTTC |
| frr149.silent.F | 5’-AATCGTTCGTGGTGAAGCAG |
| frr149silent.R | 5’-TCAGTTCTGCTTCTTTGTCTG |
| ppa37.silent.F | 5’-TGCGGGTAAAGATCTGC |
| ppa37.silent.R | 5’-CAACCGGGTCACCGTCC |
| ppa115.silent.F | 5’-TTCTGTGATCCGTTGCCG |
| ppa115.silent.R | 5’-TTCGAGGTCTTTGTAGTGC |
| recA.ec.F | 5’-AACCCGCGTGAAAGTG |
| recA.ec.R | 5’-ATCTTTCAGCCAGGCAG |
| argC.st.del.F | 5’-AACGTTTTTCATTGTTGACAC |
| argC.st.del.R | 5’-GGGACTCACGATAGTTGA |
| thrB.ec.del.F | 5’- TGGTACTGCGCGGATATG |
| thrB.ec.del.R | 5’- AAACAGCCCCTGATTTTTG |

**Supplementary Table 4.** Components used to generate mutation cassettes.

| Strain | Target gene | Modification | 5’- and 3’- Mutation fragments | Selection cassette template plasmid | Primers used to amplify the mutation cassette from the template plasmid |
| --- | --- | --- | --- | --- | --- |
| *E. coli* K12 MG1655 | *rpoD* | C-terminal 3xFLAG | rpoD.ec.FLAG.up  rpoD.ec.FLAG.down | pASC | rpoD.ec.F  rpoD.ec.R |
| *E. coli* K12 MG1655 | *gapA* | C-terminal 3xFLAG | gapA.ec.FLAG.up  gapA.ec.FLA.down | pASC | gapA.ec.F  gapA.ec.R |
| *E. coli* K12 MG1655 | *gapA* | C-terminal 3xFLAG | gapA.ec.FLAG.up2  gapA.ec.FLAG.down2 | pTSC or pT2SC | gapA.ec.F  gapA.ec.R |
| S. *enterica* SL1344 | *gapA* | C-terminal 3xFLAG | gapA.st.FLAG.up  gapA.st.FLAG.down | pASC | gapA.st.F  gapA.st.R |
| S. enterica SL1344 | *gapA* | C-terminal 3xFLAG | gapA.st.FLAG.up2  gapA.st.FLAG.down2 | pTSC or pT2SC | gapA.st.F  gapA.st.R |
| *E. coli* K12 MG1655 | *frr* | silent mutation of 33S | frr33.silent.up  frr33.silent.down | pT2SC | frr33.silent.F  frr33.silent.R |
| *E. coli* K12 MG1655 | *frr* | silent mutation of 149S | frr149.silent.up  frr149.silent.down | pT2SC | frr149.silent.F  frr149.silent.R |
| *E. coli* K12 MG1655 | *ppa* | silent mutation of 37S | ppa37.silent.up  ppa37.silent.down or  ppa37.silent.down2 | pT2SC  or  pT2SCb | ppa37.silent.F  ppa37.silent.R |
| *E. coli* K12 MG1655 | *ppa* | silent mutation of 115S | ppa115.silent.up  ppa115.silent.down | pT2SC | ppa115.silent.F  ppa115.silent.R |
| *E. coli* K12 BW25113 | *recA 2278-5* | 3 base pair changes | recA.ec.2278-5.up3  recA.ec.2278-5.down3 | pT2ST | recA.ec.F  recA.ec.R |
| S. *enterica* SL1344 | *argC* | deletion | argC.st.del.up  argC.st.del.down | pASC | argC.st.del.F  argC.st.del.R |
| *E. coli* K12 MG1655 | *thrB* | deletion | thrB.ec.del.up  thrB.ec.del.down | pT2SK | thrB.ec.del.F  thrB.ec.del.R |

**Supplemental List of synthetic DNA fragments**

>gBlock1 (encodes a fragment of *tetR* followed by a fragment of *I-sceI*)

taaagtaaaatgccccacagcGCTGAGTGCATATAATGCATTCTCTAGTGAAAAACCTTGTTGGCATAAAAAGGCTAATTGATTTTCGAGAGTTTCATACTGTTTTTCTGTAGGCCGTGTACCTAAATGTACTTTTGCTCCATCGCGATGACTTAGTAAAGCACATCTAAAACTTTTAGCGTTATTACGTAAAAAATCTTGCCAGCTTTCCCCTTCTAAAGGGCAAAAGTGAGTATGGTGCCTATCTAACATCTCAATGGCTAAGGCGTCGAGCAAAGCCCGCTTATTTTTTACATGCCAATACAATGTAGGCTGCTCTACACCTAGC**T**TCTGGGCGAGTTTACGGGTTGTTAAACCTTCGATTCCGACCTCATTAAGCAGCTCTAATGCGCTG**T**TAATCACTTTACTTTTATCTAATCTAGACATCATTAATTCCTAATTTTTGTTGACACTCTATCATTGATAGAGTTATTTTACCActccctatcagtgatagagaaaagtgaaatgcat**atgaaaaacatca**AAAAAAACCAGGTAATGAACCTGGGTCCGAACTCTAAACTGCTGAAAGAATACAAATCCCAGctgatcgaactgaacatcgaac

- Underlined sequence shown in bold lower cases fixes the truncated I-SceI.
- Two base pairs in red fix mutations in TetR.
- Sequences that overlap with the ends of the AfeI/PvuII fragment of pKDTS are shown in lower case at the 5’- and 3’- ends.

>gBlock2 (encodes a fragment of *tetR* followed by a fragment of *I-sceI*)

taaagtaaaatgccccacagcGCTGAGTGCATATAATGCATTCTCTAGTGAAAAACCTTGTTGGCATAAAAAGGCTAATTGATTTTCGAGAGTTTCATACTGTTTTTCTGTAGGCCGTGTACCTAAATGTACTTTTGCTCCATCGCGATGACTTAGTAAAGCACATCTAAAACTTTTAGCGTTATTACGTAAAAAATCTTGCCAGCTTTCCCCTTCTAAAGGGCAAAAGTGAGTATGGTGCCTATCTAACATCTCAATGGCTAAGGCGTCGAGCAAAGCCCGCTTATTTTTTACATGCCAATACAATGTAGGCTGCTCTACACCTAGCCTCTGGGCGAGTTTACGGGTTGTTAAACCTTCGATTCCGACCTCATTAAGCAGCTCTAATGCGCTGCTAATCACTTTACTTTTATCTAATCTAGACATCATTAATTCCTAATTTTTGTTGACACTCTATCATTGATAGAGTTATTTTACCActccctatcagtgatagagaaaagtgaaatgcat**atgaaaaacatca**AAAAAAACCAGGTAATGAACCTGGGTCCGAACTCTAAACTGCTGAAAGAATACAAATCCCAGctgatcgaactgaacatcgaac

- Underlined sequence shown in bold lower cases fixes the truncated I-SceI.
- Sequences that overlap with either ends of the AfeI/PvuII fragment of pKDTS are shown in lower case at the 5’- and 3’- ends.

>gISceIdfrA (encodes the I-SceI cleavage site, *dfrA*, and a 16 bp universal primer binding site)

aggcgtatcacgaggcccttTAGGGATAACAGGGTAATCGGATAGACGGCATGCACGATTTGTAATAACAGAGTGTCTTGTATTTTTAAAGAAAGTCTATTTAATACAAGTGATTATATTAATTAACGGTAAGCATCAGCGGGTGACAAAACGAGCATGCTTACTAATAAAATGTTAACCTCTGAGGAAGAATTGTGAAACTATCACTAATGGTAGCTATATCGAAGAATGGAGTTATCGGGAATGGCCCTGATATTCCATGGAGTGCCAAAGGTGAACAGCTCCTGTTTAAAGCTATTACCTATAACCAATGGCTGTTGGTTGGACGCAAGACTTTTGAATCAATGGGAGCATTACCCAACCGAAAGTATGCGGTCGTAACACGTTCAAGTTTTACATCTGACAATGAGGACGTATTGATCTTTCCATCAATTAAAGATGCTTTAACCAACCTAAAGAAAATAACGGATCATGTCATTGTTTCAGGTGGTGGGGAGATATACAAAAGCCTGATCGATCAAGTAGATACACTACATATATCTACAATAGACATCGAGCCGGAAGGTGATGTTTACTTTCCTGAAATCCCCAGCAATTTTAGGCCAGTTTTTACCCAAGACTTCGCCTCTAACATAAATTATAGTTACCAAATCTGGCAAAAGGGTTAAgcagggcggggcgTAAcgcaggaaagaacatgtgag

- Sequences in lower case at the 5’- and 3’-ends overlap with pHA_1887_

>ISceIcat2 (encodes the I-SceI site and a modified *cat* at 3’-end)

tagggataacagggtaatcctggtgtccctgttgataccgggaagccctgggccaacttttggcgaaaatgagacgttgatcggcacgtaagaggttccaactttcaccataatgaaataagatcactaccgggcgtattttttgagttatcgagattttcaggagctaaggaagctaaaATGgagaaaaaaatcactggatataccaccgttgatatatcccaatggcatcgtaaagaacattttgaggcatttcagtcagttgctcaatgtacctataaccagaccgttcagctggatattacggcctttttaaagaccgtaaagaaaaataagcacaagttttatccggcctttattcacattcttgcccgcctgatgaatgctcatccggaattccgtatggcaatgaaagacggtgagctggtgatatgggatagtgttcacccttgttacaccgttttccatgagcaaactgaaacgttttcatcgctctggagtgaataccacgacgatttccggcagtttctacacatatattcgcaagatgtggcgtgttacggtgaaaacctggcctatttccctaaagggtttattgagaatatgtttttcgtctcagccaatccctgggtgagtttcaccagttttgatttaaacgtggccaatatggacaacttcttcgcccccgttttcaccatgggcaaatattatacgcaaggcgacaaggtgctgatgccgctggcgattcaggttcatcatgccgtTtgtgatggcttccatgtcggcagaatgcttaatgaattacaacagtactgcgatgagtggcaAGGAGGTgcATAacgcaggaaagaacatgtgag

- Sequences in lower case at the 3’-ends overlap with pHA_1887_.
- Four bases in red change the 3’-end of *cat* to provide a better ribosomal binding site for the downstream gene.

>STTS (encodes **two transcriptional terminators** surrounded by spacers and the I-SceI site)

aggcgtatcacgaggcccttatctcaagagtggcagcggttctgttaagtaactgaacccaatgtcgttagtgacgcttacc**cgcaaaaaaccccgcttcggcggggttttttcgc**tcttaagaggtcactgacctaaca**aaaaaaaaaccccgcccctgacagggcggggtttttttt**ggtcttgagtggcagagtcagttatcgcgagcagtatgtaagtagatcctcagtgtcagctagggataacagggtaat

- Sequence in lower case at the 5’-end overlaps with pHA_1887_.

>STS (encodes **a transcriptional terminator** surrounded by spacers and the I-SceI site)

aggcgtatcacgaggcccttatctcaagagtggcagcggtcttgagtggcagcggcggtatacggcagcggtatgtaactagctcctcagtggcagcggtgaggaggc**aaaaaaaaaccccgcccctgacagggcggggtttttttt**aggttctgttaagtaactgaacccaatgtcgttagtgacgcttacctcttaagaggtcactgacctaacatagggataacagggtaat

- Sequence in lower case at the 5’-end overlaps with pHA_1887_

**Sequences of Mutation Fragments**

The two mutation fragments used for each target modification described in the text are given below. Sequences that overlap the vector or selection cassette sequences required for Gibson assembly are shown in lower case at the 5’- and 3’-ends of the fragment. HR3 is highlighted in yellow. Synonymous codons are shown in blue. Some mutation fragments were assembled with pSMART-HC-Amp rather than with pHA_1877_ to generate mutation template plasmids. (These fragments are marked with superscript S at the end of the name of the fragment).

**1. *E. coli rpoD* 3XFLAG**

*3X FLAG sequence is underlined and the I-SceI site is highlighted. The stop codon of the target gene is shown in lower case.

>rpoD.ec.FLAG.up^S^

atatcaagcttgaattcgttAGCAAAAGTTCTGCGTATGCGTTTCGGTATCGATATGAACACCGACTACACGCTGGAAGAAGTGGGTAAACAGTTCGACGTTACCCGCGAACGTATCCGTCAGATCGAAGCGAAGGCGCTGCGCAAACTGCGTCACCCGAGCCGTTCTGAAGTGCTGCGTAGCTTCCTGGACGATGACTACAAAGACCATGACGGTGATTATAAAGATCATGATATCGA**CTACAAGGATGACGATGACAAGtaaTCGGTAGGCCGGATCAGGCGTTACG**tagggataacagggtaat

>rpoD.ec.FLAG.down^S^

gcagggcggggcgtaa**CTACAAGGATGACGATGACAAGtaaTCGGTAGGCCGGATCAGGCGTTACG**CCGCACCCGGCACTAGGCCCTCTGCACAAACGCCACCTTTTCGGTGGCGTTTTTTATCGCCCACGCACTACCAGCGCCTGGTCCAGCTCGCGATACGCTTCAACCAGTTTCTCCAGTGAAACGCGACTTAAACCGCTGGGATTTGGCAGCgacgaattctctagatatcg

**2. *E. coli* K-12 *thrB* deletion**

>thrB.ec.del.up

aggcgtatcacgaggcccttCGAAGTGGATGGTAATGATCCGCTGTTCAAAGTGAAAAATGGCGAAAACGCCCTGGCCTTCTATAGCCACTATTATCAGCCGCTGCCGTTGGTACTGCGCGGATATGGTGCGGGCAATGACGTTACAGCTGCCGGTGTCTTTGCTGATCTGCTACGTACCCTCTCATGGAAGTTAGGAGTCTGACATGAAACTCTACAATatctcaagagtggcagcggt

>thrB.ec.del.down

gcagggcggggcgtaaAGTTAGGAGTCTGACATGAAACTCTACAATCTGAAAGATCACAACGAGCAGGTCAGCTTTGCGCAAGCCGTAACCCAGGGGTTGGGCAAAAATCAGGGGCTGTTTTTTCCGCACGACCTGCCGGAATTCAGCCTGACTGAAATTGATGAGATGCTGAAGCTGGATTTTGTCACCCGCAGTGCGAAGATCCTCTCGGCGTTcgcaggaaagaacatgtgag

**3.  *S. enterica* SL1344 *argC* deletion**

*The I-SceI site is highlighted.

>argC.st.del.up^S^

atatcaagcttgaattcgttTCTTCGGTTGCGCTTATCGACGGTGTGGCAATCAAAGCGCGGTAAATCTCGATAAATGGCGGTAAAACGTTTTTCATTGTTGACACACCTCAGGTCATGATAGTATCAATATTCATGCATTAATTATGAATAAAAATACATTAACGTTGAGCATAAAGGAACCCGATGATGAATCCATTAATTATCAAGCTGGGTGGCGTtagggataacagggtaat

>argC.st.del.down^s^

gcagggcggggcgtaaTAAAAATACATTAACGTTGAGCATAAAGGAACCCGATGATGAATCCATTAATTATCAAGCTGGGTGGCGTATTACTGGATAGCGAAGAGGCTCTGGAACGTCTTTTTACCGCGCTGGTCAACTATCGTGAGTCCCATCAGCGTCCGCTGGTGATTGTTCACGGCGGCGGTTGCGTGGTGGATGAGCTGATGAAAGGGCTTAgacgaattctctagatatcg

**4. *E. coli gapA* 3XFLAG**

*3X FLAG sequence is underlined and the I-SceI site is highlighted. The stop codon of the target gene is shown in lower case in HR3.

>gapA.ec.FLAG.up^S^

atatcaagcttgaattcgttCTACACCGAAGATGACGTAGTATCTACCGATTTCAACGGCGAAGTTTGCACTTCCGTGTTCGATGCTAAAGCTGGTATCGCTCTGAACGACAACTTCGTGAAACTGGTATCCTGGTACGACAACGAAACCGGTTACTCCAACAAAGTTCTGGACCTGATCGCTCACATCTCCAAAGACTACAAAGACCATGACGGTGATTATAAAGATCATGATATCGA**CTACAAGGATGACGATGACAAGtaaGTTGAGATGACACTGTGATCTAAAA**tagggataacagggtaat

>gapA.ec.FLAG.down^S^

gcagggcggggcgtaa**CTACAAGGATGACGATGACAAGtaaGTTGAGATGACACTGTGATCTAAAA**AGAGCGACTTCGGTCGCTCTTTTTTTTACCTGATAAAATGAAGTTAAAGGACTGCGTCATGATTAAGAAAATTTTTGCCCTTCCGGTCATCGAACAAATCTCCCCTGTCCTCTCCCGTCGTAAACTGGATGAACTGGACCTCATTGTGGTgacgaattctctagatatcg

>gapA.ec.FLAG.up2

aggcgtatcacgaggcccttCTACACCGAAGATGACGTAGTATCTACCGATTTCAACGGCGAAGTTTGCACTTCCGTGTTCGATGCTAAAGCTGGTATCGCTCTGAACGACAACTTCGTGAAACTGGTATCCTGGTACGACAACGAAACCGGTTACTCCAACAAAGTTCTGGACCTGATCGCTCACATCTCCAAAGACTACAAAGACCATGACGGTGATTATAAAGATCATGATATCGA**CTACAAGGATGACGATGACAAGtaaGTTGAGATGACACTGTGATCTAAAA**atctcaagagtggcagcggt

>gapA.ec.FLAG.down2

gcagggcggggcgtaa**CTACAAGGATGACGATGACAAGtaaGTTGAGATGACACTGTGATCTAAAA**AGAGCGACTTCGGTCGCTCTTTTTTTTACCTGATAAAATGAAGTTAAAGGACTGCGTCATGATTAAGAAAATTTTTGCCCTTCCGGTCATCGAACAAATCTCCCCTGTCCTCTCCCGTCGTAAACTGGATGAACTGGACCTCATTGTGGTcgcaggaaagaacatgtgag

**5. *Salmonella gapA* 3XFLAG**

*3X FLAG sequence is underlined and the I-SceI site is highlighted. The stop codon of the target gene is shown in lower case in HR3.

>gapA.st.FLAG.up^S^

atatcaagcttgaattcgttTTACACCGAAGACGACGTTGTATCTACCGATTTCAACGGTGAAGTATGCACTTCCGTGTTCGATGCTAAAGCAGGCATCGCGCTGAACGACAACTTCGTGAAACTGGTCTCCTGGTACGATAACGAAACCGGTTACTCCAACAAAGTACTGGACCTGATTGCTCACATCTCCAAAGACTACAAAGACCATGACGGTGATTATAAAGATCATGATATCGA**CTACAAGGATGACGATGACAAGtaaGTTGAGATGACACAGTCATTGGTAA**tagggataacagggtaat

>gapA.st.FLAG.down^S^

gcagggcggggcgtaa**CTACAAGGATGACGATGACAAGtaaGTTGAGATGACACAGTCATTGGTAA**GAGCGACTCAGGTCGCTCTTTTTTTTGCTTAAAGATATACCCGTCATACTTCAAGTTGCAGGTGTGTTGGCTATGCTTTCTCACCCGAATCACTGACGGAAGTCGAACTTATCGGGATGAATCAGGGATGTCCATGTCCCTGGCCGGAGAgacgaattctctagatatcg

>gapA.st.FLAG.up2

aggcgtatcacgaggcccttTTACACCGAAGACGACGTTGTATCTACCGATTTCAACGGTGAAGTATGCACTTCCGTGTTCGATGCTAAAGCAGGCATCGCGCTGAACGACAACTTCGTGAAACTGGTCTCCTGGTACGATAACGAAACCGGTTACTCCAACAAAGTACTGGACCTGATTGCTCACATCTCCAAAGACTACAAAGACCATGACGGTGATTATAAAGATCATGATATCGA**CTACAAGGATGACGATGACAAGtaaGTTGAGATGACACAGTCATTGGTAA**atctcaagagtggcagcggt

>gapA.st.FLAG.down2

gcagggcggggcgtaa**CTACAAGGATGACGATGACAAGtaaGTTGAGATGACACAGTCATTGGTAA**GAGCGACTCAGGTCGCTCTTTTTTTTGCTTAAAGATATACCCGTCATACTTCAAGTTGCAGGTGTGTTGGCTATGCTTTCTCACCCGAATCACTGACGGAAGTCGAACTTATCGGGATGAATCAGGGATGTCCATGTCCCTGGCCGGAGAcgcaggaaagaacatgtgag

**6. Silent mutation of 33S of Frr**

* Modified codon in red.

>frr33.silent.up

aggcgtatcacgaggcccttATATGTTTAATCAGGGCTATACTTAGCACACTTCCACTGTGTGTGACTGTCTGGTCTGACTGAGACAAGTTTTCAAGGATTCGTAACgtgATTAGCGATATCAGAAAAGATGCTGAAGTACGCATGGACAAATGCGTAGAAGCGTTCAAAACCCAAATCAGCAAAATAC**GCACGGGTCGTGCTAGCCCCAGCCTGCTGGA**atctcaagagtggcagcggt

>frr33.silent.down

*** (*frr* start codon)

gcagggcggggcgtaaTGATCTCTGACATTCGTAAGGACGCGGAGGTGCGTATGGATAAGTGTGTTGAGGCATTTAAGACGCAGATTTCTAAGATCC**GCACGGGTCGTGCTAGCCCCAGCCTGCTGGA**TGGCATTGTCGTGGAATATTACGGCACGCCGACGCCGCTGCGTCAGCTGGCAAGCGTAACGGTAGAAGATTCCCGTACACTGAAAATCAACGTGTTTGATCGTTCAATGTCTCCGGCCGTTGAAAAAGCGATTATGGCGTCCGATCTTGGCCTGAACCCGAACTCTGCGcgcaggaaagaacatgtgag

**7. Silent mutation of 149S of Frr**

* Modified codon in red.

>frr149.silent.up

aggcgtatcacgaggcccttGGCCTGAACCCGAACTCTGCGGGTAGCGACATCCGTGTTCCGCTGCCGCCGCTGACGGAAGAACGTCGTAAAGATCTGACCAAAATCGTTCGTGGTGAAGCAGAACAAGCGCGTGTTGCAGTACGTAACGTGCGTCGTGACGCGAACGACAAAGTGAAAGCACTGTTGA**AAGATAAAGAGATCTCTGAAGACGACGATCG**CCGCAGCCAAGATGACGTGCAAAAGTTAACCGACGCGGCCATTAAAAAGATCGAGGCAGCCTTGGCCGATAAGGAGGCGGAGTTAATGCAATTTatctcaagagtggcagcggt

>frr149.silent.down

gcagggcggggcgtaa**AAGATAAAGAGATCTCTGAAGACGACGATCG**CCGTTCTCAGGACGATGTACAGAAACTGACTGATGCTGCAATCAAGAAAATTGAAGCGGCGCTGGCAGACAAAGAAGCAGAACTGATGCAGTTCtgaTTTCTTGAACGACAAAAACGCCGCTCAGTAGATCCTTGCGGATCGGCTGGCGGCGTTTTGCTTTTTATTCTGcgcaggaaagaacatgtgag

**8. Silent mutation of 37S of Ppa**

* Modified codon in red.

>ppa37.silent.up

aggcgtatcacgaggcccttCAAGCGAAGACATTCGGCGCGAGTTGGCTATAATACTCGGCACTTGTTTGCCACATATTTTTAAAGGAAACAGACatgAGCTTACTCAACGTCCCTGCGGGTAAAGATCTGCCGGAAGACATCTACGTTGTTATTGAGATCCCGGCTAACGCAGATCCGATCAAATAC**GAAATCGACAAAGAGTCTGGCGCACTGTTCGT**atctcaagagtggcagcggt

>ppa37.silent.down

*** (*ppa* start codon)

gcagggcggggcgtaaTGTCGCTGTTGAATGTGCCCGCCGGCAAGGACTTGCCTGAGGATATTTATGTAGTGATCGAAATTCCAGCGAATGCTGACCCTATTAAGTAT**GAAATCGACAAAGAGTCTGGCGCACTGTTCGT**TGACCGCTTCATGTCCACCGCGATGTTCTATCCATGCAACTACGGTTACATCAACCACACCCTGTCTCTGGACGGTGACCCGGTTGACGTACTGGTCCCAACTCCGTACCCGCTGCAGCCGGGTTCTGTGATCCGTTGCCGTCCGGTTGGCGTTCTGAAAATGACCGACcgcaggaaagaacatgtgag

>ppa37.silent.down2

*** (*ppa* start codon)

gcaaggaggtgcataaTGTCGCTGTTGAATGTGCCCGCCGGCAAGGACTTGCCTGAGGATATTTATGTAGTGATCGAAATTCCAGCGAATGCTGACCCTATTAAGTAT**GAAATCGACAAAGAGTCTGGCGCACTGTTCGT**TGACCGCTTCATGTCCACCGCGATGTTCTATCCATGCAACTACGGTTACATCAACCACACCCTGTCTCTGGACGGTGACCCGGTTGACGTACTGGTCCCAACTCCGTACCCGCTGCAGCCGGGTTCTGTGATCCGTTGCCGTCCGGTTGGCGTTCTGAAAATGACCGACcgcaggaaagaacatgtgag

**9. Silent mutation of 115S of Ppa**

* Modified codon in red.

>ppa115.silent.up

aggcgtatcacgaggcccttTGCAACTACGGTTACATCAACCACACCCTGTCTCTGGACGGTGACCCGGTTGACGTACTGGTCCCAACTCCGTACCCGCTGCAGCCGGGTTCTGTGATCCGTTGCCGTCCGGTTGGCGTTCTGAAAATGACCGACGAAGCCGGTGAAGATGCGAAACTGGTTGCGGTTC**CGCACAGCAAGCTGTCTAAAGAATACGATCA**TATCAAGGATGTGAATGACTTACCGGAGCTTTTGAAGGCTCAGATTGCCCATTTTTTTGAACATTATAAGGATTTGGAGAAGGGGAAATGGGTTAAAGTCGAGGGGTGGGAGAATGCCGAGGCGGCCAAGGCGGAGATTGTGGCAAGCTTTGAACGTGCCAAAAACAAGTAAatctcaagagtggcagcggt

>ppa115.silent.down

gcagggcggggcgtaa**CGCACAGCAAGCTGTCTAAAGAATACGATCA**CATTAAAGACGTTAACGATCTGCCTGAACTGCTGAAAGCGCAAATCGCTCACTTCTTCGAGCACTACAAAGACCTCGAAAAAGGCAAGTGGGTGAAAGTTGAAGGTTGGGAAAACGCAGAAGCCGCTAAAGCTGAAATCGTTGCCTCCTTCGAGCGCGCAAAGAATAAAcgcaggaaagaacatgtgag

**10. *E. coli* *recA2278-5* mutation**

*In this case, mutated bases (red) are not in HR3.

>recA.ec.2278-5.up

aggcgtatcacgaggcccttATTCTACGCCTCTGTTCGTCTCGACATCCGTCGTATCGGCGCGGTGAAAGAGGGCGAAAACGTGGTGGGTAGCGAAACCCGCGTGAAAGTGGTGAAGAACAAAATCGCTGCGCCGTTTAAACAGGCTGAATTCCAGATCCTCTAC**GGCGAAGGTATCAACTTCTACGGCGAACTG**GTCGATTTAGGTGTCAAGGAAAAATTGATTGAAAAGGCTGGGGCATGGTATAGTTATAAGGGCGAAAAAATTGGCCAAGGCAAGGCCAACGCCACCGCGTGGCTTAAGGACAATCCAGAGACTGCCAAGGAAATTGAAAAAAAGGTGCGCGAACTGTTGTTGTCTAATCCAAATAGTACCCCAGACTTTTCGGTTGACGACTCTGAGGGTGTTGCGGAGACAAATGAGGACTTCTAAatctcaagagtggcagcggt

>recA.ec.2278-5.down

gcagggcggggcgtaa**GGCGAAGGTATCAACTTCTACGGCGAACTG**tTTGACCTGacCGTAAAAGAGAAGCTGATCGAGAAAGCAGGCGCGTGGTACAGCTACAAAGGTGAGAAGATCGGTCAGGGTAAAGCGAATGCGACTGCCTGGCTGAAAGATAACCCGGAAACCGCGAAAGAGATCGAGAAGAAAGTACGTGAGTTGCTGCTGAGCAACCCGAACTCAACGCCGGATcgcaggaaagaacatgtgag
